# Monthly variation of fatty acids, lipid quality index and metal content of *Pontastacus leptodactylus* (Eschscholtz, 1823) in Atikhisar Dam Lake (Çanakkale, Türkiye)

**DOI:** 10.1007/s11356-024-32858-1

**Published:** 2024-03-19

**Authors:** Selçuk Berber, Sefa Acarlı, Barış Bayraklı, Semih Kale, Bayram Kızılkaya, Pervin Vural, Deniz Acarlı

**Affiliations:** 1 Department of Marine and Inland Water Sciences, Faculty of Marine Sciences and Technology, Çanakkale Onsekiz Mart University, 17020 Çanakkale, Türkiye; 2 Department of Aquaculture, Faculty of Marine Sciences and Technology, Çanakkale Onsekiz Mart University, 17020 Çanakkale, Türkiye; 3 Department of Fisheries, Vocational School, Sinop University, Sinop, 57000 Türkiye; 4 Department of Fishing and Fish Processing Technology, Faculty of Marine Sciences and Technology, Çanakkale Onsekiz Mart University, 17020 Çanakkale, Türkiye; 5 Department of Aquaculture, Bayramiç Vocational School, Çanakkale Onsekiz Mart University, 17700 Çanakkale, Türkiye; 6 Department of Motor Vehicles and Transportation Technologies, Underwater Technology Program, Vocational School of Maritime Technologies, Çanakkale Onsekiz Mart University, 17020 Çanakkale, Türkiye

**Keywords:** EFA, Health risks, Lipid quality index, Metal content, *Pontastacus leptodactylus*

## Abstract

This study aims to investigate the metal content, fatty acid composition, lipid quality, and potential health risks of *Pontastacus leptodactylus* crayfish inhabiting Atikhisar Dam Lake. The research covers a 12-month period and includes both male and female individuals. The study investigated the metal content of crayfish specimens. In female individuals, the metal concentrations were ranked as Fe > Zn > Al > Cu > Mn > Se > As > Hg > Cd > Pb, while in male individuals, the ranking was Fe > Al > Zn > Cu > Mn > Se > As > Hg > Pb > Cd. The results demonstrate that Atherogenicity Index (AI) values for both genders range between 0.21 and 0.31, and Thrombogenicity Index (TI) values fall within 0.14 and 0.20. This indicates that crayfish meat is composed of healthy and high-quality fatty acids. In male individuals, omega-3 values range from 25.28 ± 0.380% to 28.34 ± 0.430%, and in female individuals, they vary from 22.98 ± 0.195% to 28.73 ± 0.871%. These findings underscore the absence of significant health risks associated with mercury levels in crayfish meat. Monthly meal calculations reveal that consuming female crayfish at an average of 4.35 servings per month for adults and 2.24 servings per month for children presents no health hazards. Similarly, the consumption of crayfish meat at an average of 5.29 servings per month for adult males and 2.72 servings per month for male children is deemed safe for health. Based on these results, the lipid quality of both male and female individuals from this species is found to be beneficial, as confirmed by risk–benefit assessments.

## Introduction

*Astacus leptodactylus*, commonly known as Turkish crayfish, the Danube crayfish, Galician crayfish, European crayfish, or narrow-clawed crayfish, was taxonomically reclassified as "*Pontastacus leptodactylus*" in a revision conducted in 2017 (Crandall and De Grave 2017). *P. leptodactylus* is a native species found in both freshwater and brackish environments and holds significant commercial importance for the fisheries and aquaculture industries (Berber et al. 2020; Boštjančić et al. 2021). The natural habitats of crayfish encompass rivers, swamps, shallow lakes, and ponds (Kumlu 2001). It is naturally distributed in 27 countries, including Türkiye, Ukraine, Southwest Russia, the Baltic, and the Caspian Sea river channel systems, as well as Kazakhstan, Belarus, Slovakia, Bulgaria, Romania, and Hungary. Furthermore, it has been introduced into lakes and canals in the Czechia, Poland, Germany, Finland, Denmark, the Netherlands, the United Kingdom, Lithuania, Latvia, France, Switzerland, Austria, Spain, and Italy (Skurdal and Taugbøl 2002). In Türkiye, its distribution extends to Northern Anatolia and Thrace, as well as Central and Western Anatolia (Holthuis 1961; Berber et al. 2020).

The maximum size of females and males is reported to be 183 mm (Bök et al. 2013) The species is cold-water adapted, and its breeding season commences in the fall when water temperatures drop. Mature individuals reproduce once a year, exhibiting limited productivity, and have a long embryonic development period lasting 6 to 9 months (Reynolds et al. 1992; Berber and Mazlum 2009).

In Türkiye, records of crayfish production date back to 1965, with a reported production volume of 270 tons. The highest crayfish production was achieved in 1984, reaching 7.937 tons. However, in the past decade, fluctuations in fishing production have been observed, and the Turkish crayfish production in 2021 was recorded at 2.022 tons (TUIK 2022).

Lipids, one of the fundamental components of food, not only serve as a high-energy source but also play crucial roles in nutrition and health, depending on the properties of the fatty acids (Bayrakli 2021a; Vural 2022). Certain lipids, which are essential for various physiological activities, can not be synthesized by humans (Yildiz et al. 2021). The significance of fatty acids, such as EPA (eicosapentaenoic acid, 20:5n-3) and DHA (docosahexaenoic acid, 22:6n-3), lies in their positive effects on autoimmune and inflammatory bowel diseases, cancer prevention, brain-related disorders, immune system functioning, and overall enhancement of life quality (Bayraklı and Duyar 2019). Polyunsaturated fatty acids (PUFAs), particularly omega-3 fatty acids, are abundant in marine and inland species, such as fishes (Gil and Gil 2015), mollusc, echinoderms (Kabeya et al. 2017), and crustaceans (Tsape et al. 2010), which are essential food sources for humans. Consequently, these species, with their nutritional importance, also hold significant commercial and economic value.

In contrast, research on crayfish in Türkiye has primarily focused on the taxonomy, biology, bioecology, and fishing of the species (Holthuis 1961; Akhan et al. 2014). However, studies on the biochemical composition and nutritional value of the species remain relatively limited (Mazlum et al. 2019).

Crustaceans living in wetlands can be exposed to metal pollution, which can have serious effects on human health. Particularly, heavy metals such as arsenic (As), lead (Pb), mercury (Hg), and Cd, along with insecticides, industrial waste, and environmental pollutants, can reach wetlands and be transferred to humans through the food chain. These heavy metals, especially toxic elements like As, Pb, Hg, and Cd, are known to have adverse effects on human health (Yildiz et al. 2023; Acarlı et al. 2023). The ratio between Hg and selenium (Se) is especially crucial in terms of human health. Hg is a toxin originating from environmental pollution sources and can exhibit toxic effects through biological accumulation. However, the presence of Se can mitigate the negative effects of Hg. The balance of the Hg/Se ratio can reduce or enhance toxic effects. Therefore, evaluating the Hg/Se ratio in metal analyses and emphasizing this ratio in health risk assessments is essential.

This research aims to monitor the seasonal variations in metal and fatty acid concentrations in *P. leptodactylus* species. Fish consumption is an important dietary source for human health; however, environmental factors such as metal pollution in aquatic environments can affect the quality of fish products. The results of this study aim to provide information regarding the level of metal pollution and fatty acid quality in *P. leptodactylus*, with implications for consumer health.

## Materials and methods

**Fig. 1:**
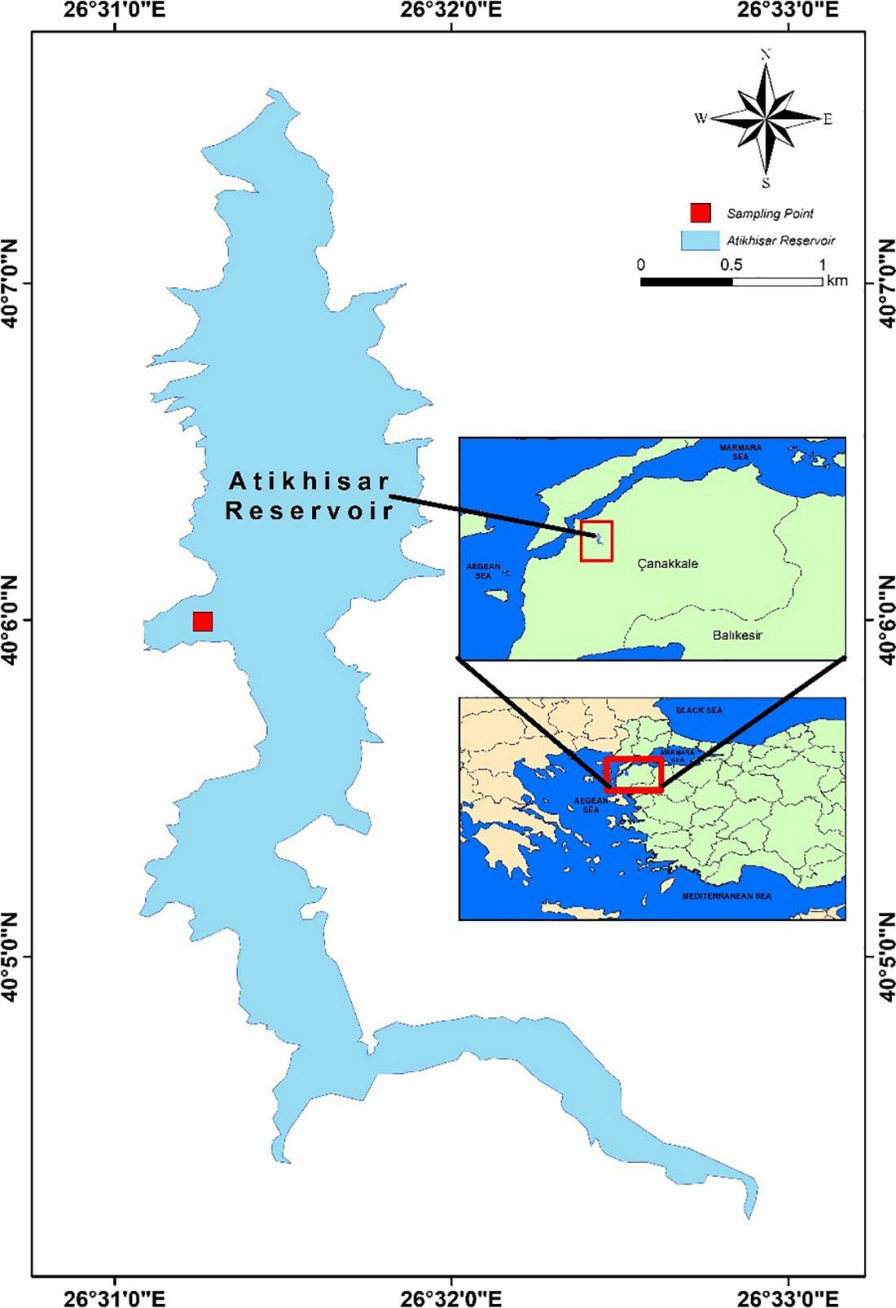
Location of Atikhisar Dam Lake

The research material utilized in this study was *P. leptodactylus*, a crayfish species belonging to the Astacidae family, found in Atikhisar Dam Lake (Fig. 1). To collect crayfish samples, a single-entrance, directed fishing gear called "pinter" with two baskets was employed. The pinter nets used in this study were 5-casqued, and a guiding net was positioned between the two pinters.

### Sampling area

Atikhisar Dam Lake is situated in the central district of Çanakkale province. The dam is approximately 11 km southeast of the city, located south of Atikhisar Castle and along the Çanakkale-Çan road. It was constructed on the Sarıçay River. The field study was planned to be conducted monthly for a period of 12 months at Atikhisar Dam Lake in Çanakkale, Türkiye. The study spanned from July 2020 to June 2021. The pinters were strategically placed in hunting-suitable sections of the lake, and regular checks were carried out. Both 30 female and 30 male (≥ 10 cm in length) were collected to do analysis during the each sampling time. The crayfish were then placed in styrofoam boxes and transported to the biochemistry laboratory of the Faculty of Marine Sciences and Technology at Çanakkale Onsekiz Mart University on the same day for analysis and measurements. The abdomen meat was dissected to be used in all analyzes.

### Metal content analysis

In the ICP experiments, 0.5 g of crayfish sample was digested in quartz vessels with 3.9 ml of HNO_3_ (65%) using a microwave digestion unit (Cem Discover SPD). The microwave digestion followed a ramp-time of 4–5 min with a hold-time of 3 min at 190 ℃. The ramp-time at 60 ℃ was 5 min maximum, with a hold-time of 3 min. After cooling to room temperature, 14 ml of distilled water were added to the digested samples. The crayfish extracts were analyzed for the presence of As, Al, Cu, Fe, Mn, Hg, Cd, Pb, and Zn using ICP-MS Optical Emission Spectrophotometer, based on NMKL 186 (2007) (Association of Official Analytical Chemists with TS EN ISO IEC 17025 AB-0744-T references number). The detection limits for ICP-OES are presented in Table 1. The certificated reference substance (NRCC-DORM-2 Dogfish Muscle) was employed as a standard for calibration verification.

**Table 1:** Monthly gender-based metal concentrations in the species *Pontastacus leptodactylus* (kg mg^−1^ ww)

*	**	Al			As			Cd			Cu			Fe			Hg			Mn			Pb			Se			Zn		
1	♀	9.00	±	0.258^c^	0.26	±	0.013^e^	0.03	±	0.001^ab^	14.46	±	0.484^cde^	18.92	±	0.609^c^	0.06	±	0.001^ cd^	2.25	±	0.047^c^	0.003	±	0.000^a^	0.35	±	0.021^abc^	18.52	±	0.523^ab^
	♂	10.19	±	0.420^b^	0.26	±	0.008^d^	0.02	±	0.005^ab^	12.42	±	0.348^bc^	11.96	±	0.362^b^	0.03	±	0.000^a^	2.78	±	0.090^d^	0.003	±	0.000^b^	0.34	±	0.020^a^	17.82	±	0.673^a^
2	♀	5.66	±	0.109^a^	0.23	±	0.012^ cd^	0.02	±	0.007^a^	13.24	±	0.111^bc^	10.22	±	0.139^a^	0.06	±	0.001^c^	1.17	±	0.056^a^	0.017	±	0.001^e^	0.32	±	0.022^a^	19.23	±	0.312^bc^
	♂	2.51	±	0.042^a^	0.21	±	0.007^ab^	0.01	±	0.007^ab^	10.20	±	0.136^a^	36.85	±	0.136^d^	0.05	±	0.002^de^	0.87	±	0.015^a^	0.017	±	0.001^d^	0.36	±	0.019^ab^	20.12	±	0.292^ cd^
3	♀	27.62	±	0.898^i^	0.26	±	0.004^e^	0.02	±	0.001^ab^	15.75	±	0.109^f^	23.05	±	0.144^d^	0.08	±	0.001^ g^	3.91	±	0.139^ h^	0.012	±	0.001^ cd^	0.34	±	0.040^ab^	18.54	±	0.265^ab^
	♂	55.00	±	2.901^ h^	0.35	±	0.003^ g^	0.04	±	0.006^b^	15.88	±	0.112^ g^	123.03	±	1.470^ g^	0.10	±	0.001^ h^	4.97	±	0.028^ g^	0.078	±	0.001^ h^	0.38	±	0.006^abc^	19.50	±	0.414^bc^
4	♀	33.70	±	0.756^ k^	0.23	±	0.005^d^	0.03	±	0.011^ab^	15.65	±	0.479^f^	26.86	±	0.686^e^	0.08	±	0.001^ g^	2.59	±	0.008^d^	0.012	±	0.002^d^	0.42	±	0.027^ cd^	19.12	±	0.490^bc^
	♂	51.62	±	0.639^ g^	0.25	±	0.006^ cd^	0.02	±	0.018^ab^	11.97	±	0.049^b^	38.10	±	0.211^d^	0.10	±	0.002^i^	2.11	±	0.034^b^	0.023	±	0.000^e^	0.34	±	0.024^a^	21.17	±	0.124^de^
5	♀	21.58	±	0.593^ h^	0.18	±	0.006^ab^	0.02	±	0.010^ab^	14.72	±	0.119^e^	17.94	±	0.216^c^	0.06	±	0.002^d^	1.72	±	0.044^b^	0.006	±	0.001^b^	0.39	±	0.048^bc^	17.65	±	0.088^a^
	♂	47.02	±	1.405^f^	0.29	±	0.009^e^	0.03	±	0.018^ab^	12.72	±	0.581^ cd^	65.27	±	3.123^f^	0.10	±	0.001^ h^	3.21	±	0.105^e^	0.033	±	0.002^f^	0.36	±	0.010^ab^	20.15	±	0.850^ cd^
6	♀	31.52	±	0.374^j^	0.19	±	0.006^ab^	0.02	±	0.003^ab^	17.17	±	0.196^ g^	71.28	±	1.301^ g^	0.05	±	0.001^b^	7.61	±	0.047^ k^	0.021	±	0.001^f^	0.40	±	0.025^bc^	23.03	±	0.431^e^
	♂	40.58	±	1.078^e^	0.32	±	0.008^f^	0.03	±	0.011^ab^	14.50	±	0.248^f^	44.03	±	0.762^e^	0.07	±	0.001^ g^	6.28	±	0.239^ h^	0.033	±	0.001^f^	0.42	±	0.025^b^	24.64	±	0.529^ g^
7	♀	14.64	±	0.340^d^	0.19	±	0.008^ab^	0.02	±	0.010^ab^	12.52	±	0.138^ab^	43.38	±	0.745^f^	0.05	±	0.002^b^	3.31	±	0.052^f^	0.007	±	0.001^b^	0.41	±	0.030^ cd^	23.05	±	0.388^e^
	♂	11.70	±	0.409^bc^	0.29	±	0.004^e^	0.02	±	0.015^ab^	12.88	±	0.047^ cd^	26.70	±	0.084^c^	0.05	±	0.001^ef^	3.59	±	0.150^f^	0.004	±	0.001^b^	0.43	±	0.046^c^	24.28	±	0.470^ g^
8	♀	15.79	±	0.340^e^	0.24	±	0.013^d^	0.03	±	0.007^ab^	19.30	±	0.291^ h^	15.14	±	0.201^b^	0.06	±	0.001^d^	5.04	±	0.156^j^	0.049	±	0.001^i^	0.48	±	0.028^d^	26.33	±	0.614^f^
	♂	10.15	±	0.204^b^	0.22	±	0.001^b^	0.02	±	0.011^ab^	9.89	±	0.054^a^	7.63	±	0.054^a^	0.05	±	0.001^ cd^	2.20	±	0.070^bc^	0.072	±	0.001^ g^	0.36	±	0.006^ab^	20.88	±	0.084^d^
9	♀	16.84	±	0.301^f^	0.18	±	0.009^a^	0.02	±	0.009^ab^	12.08	±	0.509^a^	16.15	±	0.654^b^	0.04	±	0.000^a^	3.56	±	0.073^ g^	0.012	±	0.001^ cd^	0.38	±	0.033^abc^	19.20	±	0.916^bc^
	♂	16.05	±	0.529^d^	0.20	±	0.008^a^	0.02	±	0.014^ab^	10.14	±	0.273^a^	42.42	±	1.020^e^	0.04	±	0.001^b^	3.72	±	0.148^f^	0.006	±	0.001^c^	0.38	±	0.051^abc^	20.72	±	0.315^d^
10	♀	17.34	±	0.161^ g^	0.20	±	0.006^bc^	0.02	±	0.009^ab^	14.55	±	0.389^de^	19.32	±	0.619^c^	0.07	±	0.001^e^	4.10	±	0.042^i^	0.027	±	0.001^ g^	0.35	±	0.016^abc^	20.09	±	0.689^ cd^
	♂	4.83	±	0.134^a^	0.24	±	0.006^c^	0.01	±	0.011^ab^	13.67	±	0.199^e^	7.24	±	0.102^a^	0.05	±	0.001^c^	2.90	±	0.113^d^	0.000	±	0.000^a^	0.42	±	0.013^b^	22.54	±	0.216^f^
11	♀	6.99	±	0.187^b^	0.22	±	0.010^ cd^	0.04	±	0.006^b^	17.35	±	0.569^ g^	10.01	±	0.396^a^	0.10	±	0.002^ h^	3.10	±	0.021^e^	0.010	±	0.001^c^	0.32	±	0.010^a^	22.79	±	0.835^e^
	♂	12.96	±	0.326^c^	0.22	±	0.008^b^	0.02	±	0.008^ab^	13.08	±	0.308^de^	14.61	±	0.269^b^	0.05	±	0.001^c^	2.46	±	0.044^c^	0.006	±	0.001^c^	0.40	±	0.023^abc^	22.04	±	0.396^ef^
12	♀	9.04	±	0.140^c^	0.24	±	0.008^d^	0.02	±	0.009^ab^	12.50	±	0.190^a^	22.10	±	0.433^d^	0.08	±	0.001^f^	1.72	±	0.026^b^	0.032	±	0.001^ h^	0.32	±	0.012^a^	21.14	±	0.455^b^
	♂	2.95	±	0.094^a^	0.20	±	0.004^a^	0.01	±	0.008^a^	10.22	±	0.128^a^	8.94	±	0.220^a^	0.05	±	0.001^f^	0.88	±	0.022^a^	0.001	±	0.000^a^	0.34	±	0.039^a^	18.96	±	0.276^d^

Different letters were used to indicate statistical differences. Significant differences were observed among values denoted by different letters within the same gender (*p* < 0.05). *: 1 = July 2020 to 12 = June 2021, **: Gently

### Health risk assessment

The average amount of metal accumulation (Estimated Daily Intake—EDI) for adults with an average weight of 70 kg, as described by Bayrakli (2021b), and for children with an average weight of 16 kg, according to Duyar et al. (2023), were used to determine the Target Hazard Quotient (THQ), Total Target Hazard Quotient (TTHQ), Cancer Risk (CR), and the highest daily intake (CRlim) values for cancer risk. The metal accumulation from one serving of crayfish was considered as 227 g for adults and 114 g for children, as per the references (Bayrakli 2021b). In the calculations, it was assumed that the amount of metal would not change with the cooking process.

#### Estimations of consumer health risks

In this investigation, the evaluation of consumer health risks stemming from the ingestion of metals via oral consumption is comprehensively addressed. The focus of this study involves the meticulous assessment of both non-carcinogenic and carcinogenic health risks faced by consumers exposed to these metals.

It is noteworthy to mention that the annual per capita fish consumption in the European populace stands at approximately 23 kg (Failler et al. 2007). Within the ambit of this research, a concerted effort is made to derive the estimated daily intake (EDI, expressed in mg kg^−1^ body weight/day) of metals through the consumption of crayfish meat. To accomplish this objective, the methodology outlined in the seminal works of Copat et al. (2013) and Bayrakli (2021b) is adeptly adopted. This chosen methodology exhibits a robust and well-recognized foundation for the precise estimation of daily metal intake through the ingestion of crayfish meat.

The formulation employed for this purpose is represented as follows:

$$\mathrm{EDI }= \frac{\mathrm{MC x MS}}{{\text{BW}}}$$

where;

*MC*: signifies the concentration of metals (expressed in mg kg^−1^ ww) present in crayfish meat,
*MS*: corresponds to the meal size (0.063 kg/day), denoting the average daily consumption rate of fish meat within the European Union population, and
*BW*: represents the body weight (in kg) characteristic of adults, conventionally assumed as 70 kg.

#### Non-carcinogenic risk

The assessment of non-carcinogenic risk entails a methodical evaluation whereby the level of exposure over a defined time span, typically a lifetime, is juxtaposed with a reference dose specifically recommended for the identical exposure duration. This analytical approach hinges on the utilization of the Target Hazard Quotient (THQ) to gauge non-carcinogenic risk. The THQ is determined by the ratio of the estimated daily intake (EDI) to the reference dose (RfD), the latter being a conservative threshold assumed to maintain an exposure level below which health risks for even the most susceptible populations are improbable. Should the exposure surpass this established threshold, concerns regarding potential non-cancer health ramifications are warranted.

The risk assessment pertaining to As was undertaken based on the premise that the toxic inorganic form of As constituted 3% of the aggregate content (FSA 2004). The quantification of potential risk, expounded as THQ, arising from the influence of a singular element through a solitary exposure pathway over the course of a lifetime, typically spanning 70 years, is mathematically described by the ensuing equation as delineated by the United States Environmental Protection Agency (US-EPA 2023a):

$$THQ = \frac{{\text{EDI}}}{{\text{RfD}}} x {10}^{-3}$$

where;

EDI: is the estimated daily intake (mg kg^−1^ body weight/day).
RfDrepresents: the reference doses (mg kg^−1^ day) for metals according to US-EPA (2023b).

The benchmark deemed acceptable for the Target Hazard Quotient (THQ) has been established at a value of "1" according to the US-EPA (2023a). Should the calculated THQ value fall below "1" (< 1), it signifies a presumption of negligible adverse effects on human well-being. Conversely, human exposure to metals exceeding "1" (> 1) could potentially lead to cumulative reactions within individuals (Hallenbeck 1993).

In light of this, the aggregate of THQ values is formulated into the concept of Total Target Hazard Quotients (TTHQ), which collectively contributes to the Hazard Index (HI). The Hazard Index serves as the summation of individual Hazard Quotients, wherein both Estimated Daily Intake (EDI) and Reference Dose (RfD) pertain to the same exposure duration. When the Hazard Index surpasses unity, it raises concerns about potential health-related implications. It's crucial to note that any single chemical surpassing its toxicity threshold could result in the Hazard Index exceeding unity. Moreover, even in scenarios involving multiple chemical exposures, the Hazard Index might exceed unity without any solitary chemical exposure surpassing its respective RfD.

The formulation for the non-cancer Hazard Index (HI), represented as the summation of THQ values, is established as follows, in accordance with the US EPA (2023a) guidelines and as previously described by Duyar et al. (2023) Bayrakli (2021b) for the evaluation of risks stemming from multiple elements (with "n" denoting a value of "15" in the present study):

$$\mathrm{HI }\left(\mathrm{TTHQ \Sigma n}\right)= {({\text{EDI}}}_{1}/{{\text{RfD}}}_{1}) +({{\text{EDI}}}_{2}/{{\text{RfD}}}_{2}) +({{\text{EDI}}}_{3}/{{\text{RfD}}}_{3})+ \dots \dots \dots +({{\text{EDI}}}_{n}/{{\text{RfD}}}_{n})$$

where:

EDI_n_: is the estimated intake level for the nth metal.
RfD_n_: represents the reference dose for the nth metal.

This equation encapsulates the amalgamation of multiple THQ values into a comprehensive Hazard Index, providing a quantitative assessment of potential health risks attributed to the combined influence of various metal exposures.

#### Carcinogenic risks for toxic metals

The concept of carcinogenic risks in relation to toxic metals encompasses the potential incremental likelihood of an individual developing any form of cancer throughout their lifetime due to exposure to substances with known carcinogenic properties. This risk assessment is closely linked to the individual's cumulative exposure to potential carcinogens. The Cancer Slope Factor (CSF) serves as a critical parameter in this assessment, as it directly converts the estimated daily intakes, averaged over a person's lifetime of exposure, into an incremental risk estimate that quantifies the likelihood of cancer development in an individual. The computation of Cancer Risk over a lifetime (CRR) is defined as follows:

$$CRR=EDI x CSF$$

where:

CSFdenotes the cancer slope factor for specific elements, such as As (1.5 mg kg^−1^/day), and Pb (0.0085 mg kg^−1^/day), as stipulated by the US-EPA (2023a, b).

It's important to acknowledge that although methylmercury (MeHg) has been classified as a possible human carcinogen (IARC 2012), the US-EPA has not issued a cancer slope factor for Hg. Consequently, cancer risks associated with Hg were not assessed in this study.

In the context of carcinogenic risks, the range of acceptable risk levels lies between 10^–4^ and 10^–6^. This range translates to a risk of cancer development over an individual's lifetime of 1 in 10,000 to 1 in 1,000,000, respectively. Any risk value lower than 10^–6^ is considered acceptable and negligible, whereas a risk exceeding 10^–4^ is regarded as unacceptable. Thus, the study establishes an average cancer benchmark of 10^–5^ as the threshold (US-EPA 2023a), indicating the level of risk deemed significant in this context.

#### Safe consumption limits

The determination of the maximum allowable daily consumption rate, denoted as ADC (Safe Daily Consumption Limit), is pivotal for establishing a safe threshold for daily intake. This value is calculated employing the subsequent formula, as outlined by the US-EPA (2000):

$$ADC=\frac{\mathrm{RfD x BW}}{{\text{MC}}}$$

where:

In parallel, the computation of the maximum safe weekly consumption rate, also referred to as the Allowable Weekly Consumption (AWC) in meals per week, involves converting the allowable daily consumption rate (ADC) into a unit aligned with the food meal size (MS) standard, which is set at 0.227 kg/day for adults by the US-EPA (2000). The derivation of the AWC is accomplished through the subsequent equation as derived from prior work by Yigit et al. (2018):

$$AWC= \frac{\mathrm{ ADC x }7 }{{\text{MS}}}$$

where:

#### Compensation of daily requirements for essential trace elements

The concept of compensating for daily requirements of essential trace elements assumes significance. The calculation of the percentage compensation of minimum daily requirements (CDR_min_) for these elements in humans consuming meat is executed through the equation provided by Yildiz et al. (2023):

$$CDRmin=\frac{ EDI x 100 }{EAR}$$

where:

EAR: signifies the estimated average daily requirement for a healthy human, as guided by the Institute of Medicine (IOM 2001, 2006).

#### Selenium health benefit value (Se-HBV)

The determination of the molar Se/Hg ratio is achieved through the subsequent equation, as outlined by Ralston (2008):

$$\mathrm{Molar}\;\mathrm{ratio}\left(\text{Se}/\text{Hg}\right)=\mathrm{Molar}\;\mathrm{concentration}\;\mathrm{of}\;\mathrm{Se}\left(\mathrm\mu\;\mathrm m\mathrm o\mathrm l\;\text{kg}^{-1}\right)/\mathrm{Molar}\;\mathrm{concentration}\;\mathrm{of}\;\mathrm{Hg}\left(\mathrm\mu\;\mathrm m\mathrm o\mathrm l\;\text{kg}^{-1}\right):$$

where:

The molar concentration of Se is calculated as the concentration of Se (expressed in mg kg^−1^) divided by the molar mass of Se (78.9 g mol^−1^).

The molar concentration of Hg is calculated as the concentration of Hg (expressed in mg kg^−1^) divided by the molar mass of Hg (200.59 g mol^−1^), as established by Burger and Gochfeld (2011).

The Se-HBV (Selenium-to-Mercury Binding Value) illustrates the equilibrium between Se and Hg in tissues and dietary sources. In cases where the concentration of Se in seafood is lower than that of Hg, there is an increased health risk. Conversely, higher levels of Se offer health benefits by affording protection against Hg-induced toxicity. Thus, the Se-HBV serves as a quantification tool to ascertain potential risks linked to Hg exposure or the nutritional advantages of Se consumption.

In terms of interpretation, a positive Se-HBV signifies Se-dependent health benefits. Conversely, when the result is negative, an inevitable or emerging health risk is indicated (Kaneko and Ralston 2007). The computation of Se-HBVs is facilitated by the ensuing equation, as delineated by Ulusoy et al. (2019):

$${\text{Se}}-{\text{HBV}}=\left({\text{Se}}-{\text{Hg}}\right)/\left({\text{Se}}+{\text{Hg}}\right)$$

This formulation encapsulates the calculation of Se-HBVs, allowing for the assessment of the dynamic interplay between Se and Hg concentrations and their corresponding health implications.

### Crude fat extraction

To determine the fatty acid composition of crayfish, the samples were homogenized and dried at 105 °C until they reached a constant weight in a drying oven. Crude fat extraction was performed three times on the dry tissue using the Bligh and Dyer (1959) method, which is commonly used for fat analysis. In brief, the samples were treated with methanol/chloroform. The homogenate was washed with methanol-chloroform and filtered through filter paper into a round-bottomed flask. The filtrate was then evaporated using a rotary evaporator (IKA RV10 basic) in a water bath at 60 °C. After fat separation in the round-bottomed flask, the flask was removed from the device and kept in a drying oven at 65 °C (Nüve FN500). Subsequently, it was transferred to a desiccator, cooled, and finally weighed for the final determination.

Esterification of Fatty Acids: Fatty acid analysis was performed following the AOAC (1995) standard. The crude fats of the samples were used. In summary, the crude fat samples were treated with methanolic NaOH to esterify the crude fat. Then, they were saponified by boiling in a water bath. After pouring BF_3_ reagent over the cooler, it was heated, and heptane was added. It was then cooled again without boiling and treated with saturated NaCl to form a phase. The heptane phase was transferred to a test tube and then transferred to a glass vial. Subsequently, it was injected into gas chromatography (GC) to determine the fatty acid composition.

Determination of Fatty Acid Contents by Gas Chromography (GC): Gas Chromatography (GC) was utilized to determine the fatty acid components. The system comprised an FID detector (Flame Ionization Detector), a gas chromatograph (Shimadzu, GC 2014, Japan), and an autoinjector (AOC-20i, Shimadzu, Japan). GC Solution software (Version 2.41.00 su_1) controlled the device. The chromatographic column used was FAME WAX (polyethylene glycol, 30 m length, 0.25 mm inner diameter, 0.2 µm, GC Columns Restek).

The working conditions for GC were as follows:

- Injection mode: Split ratio, split: 1/10
- Injection and detector temperature: 260 °C and 280 °C, respectively
- Carrier gas and column flow: Helium at 1.4 ml/min
- Temperature program: Starting temperature of 100 °C for 5 min, followed by an increase of 5 °C per minute from 100 °C to 150 °C, maintaining at 150 °C for 15 min, further increase of 10 °C per minute to 210 °C, and holding at 210 °C for 20 min.

Peak identification was determined using a standard (Supelco 37 Component FAMEs Mix) through gas chromatography. The data were obtained by calculating the methyl esters of fatty acids as a percentage of total fatty acids.

The percentage of fatty acid composition obtained from GC was used to calculate the amounts of fatty acids in the samples in mg/100 g portion as edible fats, following the method described by Weihrauch et al. (1977), using the fatty acid conversion factor.

### Calculation of the lipid quality indexes (LQI)

The lipid quality index was determined based on the fatty acid profile obtained from gas chromatography analyses. In this context, six different calculation methods were employed to assess the lipid quality index. These calculation methods are as follows:

- **Atherogenicity Index (AI):** [C12:0 + (4 × C14:0) + C16:0] / (n-3PUFA + n-6PUFA + MUFA) (Duyar and Bayrakli 2023; Ulbricht and Southgate 1991)
- **Thrombogenicity Index (TI):** [C14:0 + C16:0 + C18:0] / [(0.5 × C18:1) + (0.5 × sum of other MUFA) + (0.5 × n-6PUFA) + (3 × n-3PUFA) + n-3PUFA/n-6PUFA] (Duyar and Bayrakli 2023; Ulbricht and Southgate 1991)
- **Flesh-lipid quality (FLQ):** 100 * (EPA + DHA) / total fatty acids (Abrami et al. 1992)
- **Hypocholesterolemic/hypercholesterolemic ratio (h/H):** h/H = [(C18:1 + C18:2 + C18:3 + C20:3 + C20:4 + C20:5 + C22:4 + C22:5 + C22:6) / (C14:0 + C16:0)] (Duyar and Bayrakli 2023)
- **Health-promoting index (HPI):** UNSAT / [C12:0 + (C14:0 × 4) + C16:0] (Murzina et al. 2022)
- **The polyene index (PI):** (C20:5 + C22:6) / C16:0 (Lubis and Buckle 2007; Küçükgülmez et al. 2018).

### Hazard quotient for benefit–risk ratio

Gladyshev et al. (2009) have presented a formula to gauge the benefit-risk ratio associated with the consumption of marine organisms, factoring in the composition of Long-Chain Polyunsaturated Fatty Acids (LC-PUFA) as well as the presence of toxic and essential elements. This ratio is quantified through the utilization of the following equation:

$${HQ}_{EFA}=\left({R}_{EFA.}{C}_{element}\right)/\left(C.RfD.Bw\right)$$

where:

*R*_*EFA*_: signifies the recommended daily dose of essential fatty acids (EFA) for an individual (expressed in mg/day).
*C*_*element*_: denotes the concentration of the essential or toxic element (expressed in mg/kg).
*C*: represents the content of EFA, specifically Eicosapentaenoic Acid (EPA) and Docosahexaenoic Acid (DHA), within a given bivalve (expressed in mg/g).
*RfD*: stands for the reference dose for the element (expressed in mg/kg/day).
*Bw*: corresponds to the average body weight of an adult, conventionally considered as 70 kg.

In interpreting the calculated values, an HQEFA (Health-Quotient for Essential Fatty Acids) less than 1 suggests a health benefit resulting from the consumption of bivalves, whereas an HQEFA greater than 1 indicates a potential health risk (Gladyshev et al. 2009).

For the computation of this equation, the recommended daily dose of essential fatty acids *R*_*EFA*_ has been set at 500 mg/day as established by the European Food Safety Authority (2012). The values of reference doses *RfD* have been derived from the EPA Region III Risk-Based Concentrations summary table (US-EPA 2010), with the exception of Pb, for which the values were obtained from Hang et al. (2009). This formula enables a comprehensive assessment of the benefit-risk balance associated with the consumption of marine organisms, considering both the nutritional advantages of LC-PUFA and the potential health risks posed by the presence of certain elements.

### Statistical analysis

The data obtained from the monthly analyses were subjected to statistical analysis. The statistical significance of the data was determined using ANOVA (Analysis of Variance). Additionally, Tukey's comparison test was employed to identify significant differences between groups. The statistical analyses were conducted using the PAST computer program—version 1.95 (Hammer et al. 2001). The results of the statistical analysis were considered significant at the P < 0.05 level.

## Results and discussion

### Metal analysis results

The results of the metal study conducted on the freshwater crayfish showed different metal concentrations between female and male individuals. In female individuals, the metal concentrations were ranked as Fe > Zn > Al > Cu > Mn > Se > As > Hg > Cd > Pb, while in male individuals, the ranking was Fe > Al > Zn > Cu > Mn > Se > As > Hg > Pb > Cd (Table 1). Studies have shown significant differences in essential heavy metal concentrations between male and female crustaceans, indicating potential variations in metal accumulation based on gender (Nędzarek and Czerniejewski 2021; Salam and Hamdi 2014). Furthermore, the data obtained in this study indicated variations in metal concentrations of the same elements between male and female individuals across different months. This observation suggests that seasonal factors and environmental variables might influence the metal concentrations. Moreover, significant differences in element concentrations have been observed in studies conducted in different regions, indicating that the environmental conditions in various habitats of the freshwater crayfish have a substantial impact on its metal accumulation (Jorhem et al. 1994; Kır and Tuncay 2010; Kouba et al. 2010; Kurun et al. 2010; Tunca et al. 2013a; Aksu et al. 2014; Kuklina et al. 2014; Varol and Sünbül 2018). These findings contribute to the understanding of metal accumulation and metal concentrations in the freshwater crayfish, and they provide insights into the species' adaptation to environmental conditions.

Al, the third most abundant element in the Earth's crust, is extensively used in developing industries (Bohrer et al. 2008). High levels of Al accumulation can lead to chronic kidney diseases and neurological disorders in humans (Bondy 2016; Vural and Acarlı 2021). Al concentrations in both female and male individuals were lowest in August (5.66 ± 0.109 and 2.51 ± 0.042 mg kg^−1^ ww, respectively) and highest in male individuals in September (55.00 ± 2.901 mg kg^−1^ ww) and in female individuals in October (33.70 ± 0.756 mg kg^−1^ ww). The differences between the months were found to be statistically significant (p < 0.05). The obtained values showed similarities with the results of freshwater crayfish samples collected from Terkos Lake (Kurun et al. 2010) and Yeniçağa Lake (Tunca et al. 2013b). The Al values obtained in this study are consistent with the results obtained from freshwater crayfish samples collected from Terkos Lake and Yeniçağa Lake (Table 2). The findings of this study indicate that the Al concentrations in freshwater crayfish are below the World Health Organization's (WHO 1989) daily allowable limits, which have been set at 60 mg per day for adult individuals.

**Table 2:** Comparison of metal concentrations in crayfish *A. /eptodacty/us* in this study with those of *Astacus* genus from other freshwater bodies and global concentrations ranges for freshwater crayfish species

		Al	As	Cd	Hg	Cu	Fe	Mn	Ni	Pb	Zn	
	ww	12.53–235.10	1.80–3.50	0.014–0.042	0.16–0.52	9.89–19.30	7.23–123.03	0.87–7.60	0.032–0.166	Nd-0.036	17.65–23.32	Present paper
Keban Dam Lake			nd-1.46	nd-0.010	0.059–0.983	2.42–7.2				nd-0.080	4.35–21.69	(1)
Terkos Lake	ww	5.65–18.73		< 0.010–0.043		7.81–10.50	67.77–73.41	1.03–2.46		< 0.010		(2)
Kovada Lake				nd		0.12–4.7	nd-12.98	0.26–0.82	0.22–4.47	nd	9.19–12.98	(3)
Mostovoye Lake				< 0.010–0.026		4.18–11.19			nd–0.52	< 0.1	13.72–25.65	(4)
Swedish lakes			2.40	< 0.005		5.7		2.9	< 0.1	0.022	15	(5)
Global concentration	dw			0.001–0.11		0.59–29.4			0.03–1.02	0.012–3.186	1.1–25.5	(6)
Keban Dam Lake	ww		0.98	0.081		6.9	15.0	1.7	1.41	0.046	19.7	(7)
Lake Yeniçağa	dw	60.46–164.68	1.19–2.56			14.80–22.96-	4.09–8.67		0.24–2.22		17.76–198.90	(8)

1. Aksu et al. (2014); 2. Kurun et al. (2010); 3. Kır and Tuncay, (2010); 4. Kuklina et al. (2014); 5. Jorhem et al. (1994); 6. Kouba et al. (2010); 7. Varol and Sünbül (2018); 8. Tunca et al. (2013a, b)

As, relatively common in marine environments, is often present in seafood as a heavy metal (Moreda-Piñeiro et al. 2012). As concentrations were found to be lowest in March for both female and male individuals (0.18 ± 0.009 and 0.20 ± 0.008 mg kg^−1^ ww, respectively) and highest in September (0.26 ± 0.004 and 0.35 ± 0.003 mg kg^−1^ ww). The differences between the months were found to be statistically significant (p < 0.05). The obtained values showed similarities with previous studies on freshwater crayfish (Table 2). As accumulation levels may be species-specific, depending on the feeding region and seasonal variations. Additionally, As, as a heavy metal, can exist in organic or inorganic forms in nature (Raknuzzaman et al. 2016). The organic form is relatively less toxic, while the inorganic form is highly toxic (Sarkar and Paul 2016). International organizations such as the Department of Health and Human Services (DHHS), the International Agency for Research on Cancer (IARC), and the Environmental Protection Agency (EPA) have reported that inorganic As is carcinogenic to humans (ATSDR 2016). It has been reported that inorganic As accounts for about 1% to 10% of the total As content in seafood (Bayrakli 2021b). The Australia and New Zealand Food Standards (FSANZ 2013) reported an allowable value of 2 mg kg^−1^ ww for inorganic As, while the Ministry of Health of the People's Republic of China (MHPRC 2013) reported a value of 0.1 mg kg^−1^ ww. In this study, the total As content calculated for the freshwater crayfish species was determined to be 3% inorganic As (0.001–0.01 mg kg^−1^ ww), which is below the values reported by FSANZ (2013) and MHPRC (2013).

Cd concentrations were found to be lowest in August and highest in May for female individuals (0.02 ± 0.007 and 0.04 ± 0.006 mg kg^−1^ ww, respectively) and lowest in June and highest in September for male individuals (0.01 ± 0.008 and 0.04 ± 0.006 mg kg^−1^ ww, respectively). The differences between the months were statistically significant (p < 0.05). Hg concentrations were lowest in March and highest in May for female individuals (0.04 ± 0.0001 and 0.10 ± 0.002 mg kg^−1^ ww, respectively) and lowest in July and highest in November for male individuals (0.03 ± 0.0003 and 0.10 ± 0.001 mg kg^−1^ ww, respectively). The differences between the months were statistically significant (p < 0.05). Pb concentrations were lowest in July and highest in February for female individuals (0.003 ± 0.0003 and 0.049 ± 0.001 mg kg^−1^ ww, respectively) and lowest in April and highest in September for male individuals (0.0004 ± 0.0002 and 0.078 ± 0.001 mg kg^−1^ ww, respectively). The differences between the months were statistically significant (p < 0.05). The levels of these toxic heavy metals are in accordance with the results of previous studies on freshwater crayfish conducted in different regions (Table 2). These toxic heavy metals are known to pose potential threats to human health. Cd, due to its long-term accumulation and long half-life, can lead to serious health effects in the body, while Hg can cause genetic impairments and damage to cardiovascular and neurological systems. Pb is known to be one of the most toxic metals in living organisms. The Cd, Hg, and Pb levels found in this study were below the acceptable maximum values reported by the Turkish Food Codex (TFC 2011) and the European Commission (1881/2006). Therefore, the low levels of these heavy metals in the consumed freshwater crayfish may have a positive impact on reducing potential health risks.

In the conducted study, Cu levels in freshwater crayfish were found to vary according to both gender and months. Cu levels in female individuals were statistically significantly higher compared to male individuals (p < 0.05). Additionally, significant differences in copper levels were observed between different months (p < 0.05). In the female individuals of the sample, the lowest Cu levels were detected in March (12.08 ± 0.509 mg kg^−1^ ww), while the highest levels were observed in February (19.30 ± 0.291 mg kg^−1^ ww). For male individuals, the lowest Cu levels were found in February (9.89 ± 0.054 mg kg^−1^ ww), and the highest levels were observed in September (15.88 ± 0.112 mg kg^−1^ ww). This study has revealed the variation in copper levels in freshwater crayfish depending on gender and months. The higher Cu levels in female individuals suggest that gender may have an influence on Cu accumulation. This difference may be attributed to hormonal and metabolic changes specific to the female reproductive system. Cu is an essential element in many biological processes in crayfish and is known to play a role in enzymatic activities, particularly during the reproductive process. The fluctuations in Cu levels observed across different months could reflect the influence of environmental factors. For example, Cu concentrations in the water environment may vary seasonally, potentially affecting copper accumulation in crayfish. These findings are important in understanding the complex interactions between biological and environmental factors in freshwater crayfish. However, further research is needed, especially in studies that examine the effects of hormone levels and other potential factors on Cu accumulation in more detail. Such investigations can help us better understand this relationship.

Fe is one of the most abundant elements on Earth and is essential for the biological activities of all living organisms (Abbaspour et al. 2014). However, excessive intake of iron can increase the risk of chronic diseases. In our study, Fe levels in female individuals of the freshwater crayfish species were found to be lowest in May (10.01 ± 0.396 mg kg^−1^ ww) and highest in December (71.28 ± 1.301 mg kg^−1^ ww). For male individuals, the lowest iron levels were recorded in April (7.24 ± 0.102 mg kg^−1^ ww), while the highest levels were observed in September (123.03 ± 1.470 mg kg^−1^ ww). Statistical analysis revealed significant differences in Fe levels between different months (p < 0.05). The Fe levels in this study were significantly lower than the permissible limit of 300 mg/kg set by the World Health Organization (WHO 1989). When comparing our findings with previous studies, we observed various differences in the Fe data presented in Table 2. Some data were higher compared to the results reported in the literature, while others were in good agreement. These discrepancies may be attributed to various factors, such as geographical location, habitat characteristics, climate variations, dietary habits, and sampling periods, all of which can influence iron levels.

Mn is a necessary element for a healthy individual's nutrition, but it can exhibit toxic effects at high doses and lead to neurological disorders. In this study, Mn levels were lowest in both female and male individuals in August (1.17 ± 0.056 and 0.87 ± 0.015 mg kg^−1^ ww, respectively) and reached the highest levels in December (7.61 ± 0.047 and 6.28 ± 0.239 mg kg^−1^ ww, respectively). Significant differences in Mn levels were found between different months (p < 0.05). The manganese levels obtained in this study were below the permissible levels set by the World Health Organization (1989) (50 mg/kg), which is in line with the information presented in the literature (Table 2).

Se is known to play a role in heavy metal detoxification, but it is essential to remember that this process is dependent on multiple factors, and Se alone may not be sufficient. Se levels were lowest in female individuals in May (0.32 ± 0.010 mg kg-^1^ ww) and highest in February (0.48 ± 0.028 mg kg^−1^ ww). For male individuals, the lowest Se levels were recorded in June (0.34 ± 0.046 mg kg^−1^ ww), while the highest levels were observed in January (0.43 ± 0.046 mg kg^−1^ ww). Statistical analysis revealed significant differences in Se levels between different months (p < 0.05).

Zn plays a role in many physiological activities of living organisms (Jena and Dey 2016). Zn levels in female individuals of the freshwater crayfish species were lowest in November (17.65 ± 0.088 mg kg^−1^ ww) and highest in February (26.33 ± 0.614 mg kg^−1^ ww). For male individuals, the lowest Zn levels were recorded in July (17.82 ± 0.673 mg kg^−1^ ww), while the highest levels were observed in December (24.64 ± 0.529 mg kg^−1^ ww). Statistical analysis revealed significant differences in Zn levels between different months (p < 0.05). These findings are consistent with previous studies (Table 2).

### Fatty acids result

The results of the fatty acid analysis of the muscle tissues of freshwater crayfish during the July-June 2015 season revealed that it is a rich source of polyunsaturated fatty acids. The Unsaturated Fatty Acid (SFA) values in female individuals were lowest in October (23.25 ± 1.230%) and highest in April (27.55 ± 3.422%) (Table 3). For male individuals, the lowest SFA value was recorded in May (%29.48 ± 1.956) and the highest in May (32.29 ± 1.066%) (Table 4). The only significant difference in SFA levels between months was observed between October and March and April (p < 0.05), while the differences between other months were not statistically significant. The most dominant SFAs in both female and male individuals were C16:0 (Palmitic acid) (average %; 17.28 ± 1.525, 16.95 ± 1.568) and C18 (Stearic acid) (average %; 6.00 ± 1.215, 5.61 ± 1.187), respectively.

**Table 3:** Monthly variation of fatty acid ratios in female individuals of the species Pontastacus leptodactylus

	July 2020	August	September	October	November	December	January 2021	February	March	April	May	June
C14:0	0.53 ± 0.036	0.49 ± 0.027	0.45 ± 0.022	0.45 ± 0.033	0.43 ± 0.018	0.55 ± 0.009	0.43 ± 0.011	0.54 ± 0.122	0.53 ± 0.012	0.35 ± 0.032	0.32 ± 0.010	0.59 ± 0.074
C14:1	0.12 ± 0.017	0.10 ± 0.006	0.11 ± 0.010	0.13 ± 0.015	0.12 ± 0.026	0.10 ± 0.010	0.13 ± 0.020	0.10 ± 0.013	0.13 ± 0.009	0.13 ± 0.024	0.15 ± 0.016	0.16 ± 0.018
C15:0	0.23 ± 0.035	0.17 ± 0.086	0.17 ± 0.092	0.32 ± 0.057	0.60 ± 0.155	0.48 ± 0.003	0.45 ± 0.024	0.52 ± 0.030	0.36 ± 0.008	0.67 ± 0.001	0.38 ± 0.040	0.16 ± 0.003
C15:1	0.13 ± 0.010	0.12 ± 0.007	0.13 ± 0.012	0.15 ± 0.017	0.13 ± 0.030	0.11 ± 0.011	0.15 ± 0.023	0.11 ± 0.010	0.13 ± 0.005	0.15 ± 0.028	0.17 ± 0.019	0.08 ± 0.012
C16:0	15.91 ± 0.336	15.15 ± 0.158	14.99 ± 0.003	15.71 ± 0.754	17.72 ± 2.085	17.45 ± 0.359	17.58 ± 0.385	18.42 ± 0.983	19.61 ± 0.436	18.48 ± 0.890	17.39 ± 0.910	19.00 ± 1.018
C16:1	5.65 ± 0.133	5.26 ± 0.053	5.13 ± 0.079	5.23 ± 0.193	4.43 ± 1.000	6.15 ± 0.132	4.84 ± 0.128	4.80 ± 0.028	5.00 ± 0.111	3.34 ± 0.194	2.83 ± 0.361	5.01 ± 0.024
C17:0	0.88 ± 0.087	0.76 ± 0.005	0.81 ± 0.060	0.91 ± 0.036	0.77 ± 0.071	0.90 ± 0.000	0.77 ± 0.002	0.76 ± 0.097	0.80 ± 0.018	0.77 ± 0.037	0.79 ± 0.009	0.79 ± 0.089
C17:1	0.22 ± 0.086	0.22 ± 0.036	0.15 ± 0.034	0.22 ± 0.110	0.32 ± 0.033	0.26 ± 0.005	0.38 ± 0.095	0.27 ± 0.023	0.36 ± 0.008	0.30 ± 0.132	0.24 ± 0.011	0.32 ± 0.086
C18:0	5.01 ± 0.455	4.87 ± 0.219	4.56 ± 0.101	4.77 ± 0.327	6.79 ± 2.085	5.11 ± 0.011	5.93 ± 0.169	6.29 ± 0.334	6.10 ± 0.136	8.06 ± 0.206	8.12 ± 0.291	6.38 ± 0.217
C18:1n9	23.7 ± 41.232	24.54 ± 2.230	24.87 ± 2.600	24.89 ± 2.765	24.24 ± 2.107	22.02 ± 2.054	23.33 ± 1.641	24.71 ± 2.745	21.55 ± 1.707	25.00 ± 3.516	23.81 ± 2.328	22.55 ± 3.309
C18:2n6c	4.57 ± 0.292	4.51 ± 0.054	4.67 ± 0.214	5.02 ± 0.135	3.92 ± 0.619	4.04 ± 0.088	3.65 ± 0.055	5.09 ± 1.964	2.75 ± 0.061	3.34 ± 0.148	3.28 ± 0.081	5.31 ± 2.029
C18:3n6	1.31 ± 0.146	1.27 ± 0.024	1.23 ± 0.019	1.25 ± 0.038	1.03 ± 0.064	1.31 ± 0.034	1.09 ± 0.017	1.00 ± 0.038	0.99 ± 0.022	1.05 ± 0.058	1.04 ± 0.029	1.03 ± 0.084
C18:3n3	0.14 ± 0.022	0.10 ± 0.008	0.10 ± 0.008	0.10 ± 0.005	0.10 ± 0.025	0.11 ± 0.007	0.03 ± 0.030	0.16 ± 0.023	0.12 ± 0.003	0.09 ± 0.007	0.15 ± 0.003	0.13 ± 0.037
C20:0	0.35 ± 0.040	0.48 ± 0.080	0.45 ± 0.111	0.35 ± 0.014	0.35 ± 0.043	0.38 ± 0.032	0.38 ± 0.021	0.16 ± 0.011	0.12 ± 0.003	0.13 ± 0.014	0.13 ± 0.009	0.13 ± 0.014
C20:1n9	2.00 ± 0.305	2.06 ± 0.185	1.90 ± 0.347	1.58 ± 0.041	1.45 ± 0.115	1.39 ± 0.016	1.43 ± 0.036	1.32 ± 0.051	1.41 ± 0.031	1.10 ± 0.146	1.24 ± 0.061	1.29 ± 0.018
C20:2	1.46 ± 0.129	1.25 ± 0.050	1.23 ± 0.030	1.31 ± 0.048	1.08 ± 0.169	1.07 ± 0.006	1.08 ± 0.028	1.09 ± 0.101	0.77 ± 0.017	1.08 ± 0.043	1.07 ± 0.042	1.14 ± 0.087
C20:3n6	0.18 ± 0.002	0.18 ± 0.017	0.17 ± 0.004	0.18 ± 0.010	0.17 ± 0.032	0.14 ± 0.008	0.16 ± 0.001	0.18 ± 0.005	0.18 ± 0.004	0.15 ± 0.001	0.16 ± 0.004	0.19 ± 0.007
C21:0	0.09 ± 0.010	0.10 ± 0.014	0.12 ± 0.011	0.15 ± 0.016	0.13 ± 0.028	0.11 ± 0.011	0.14 ± 0.022	0.11 ± 0.015	0.15 ± 0.015	0.14 ± 0.027	0.17 ± 0.018	0.17 ± 0.020
C20:4n6	11.10 ± 0.159	11.66 ± 0.129	11.16 ± 0.385	11.10 ± 0.349	9.57 ± 0.734	8.29 ± 0.188	9.00 ± 0.196	8.71 ± 0.759	8.93 ± 0.199	8.92 ± 0.387	11.61 ± 0.288	9.08 ± 0.874
C20:3n3	0.25 ± 0.013	0.28 ± 0.044	0.29 ± 0.039	0.25 ± 0.001	0.24 ± 0.046	0.28 ± 0.005	0.26 ± 0.007	0.19 ± 0.035	0.22 ± 0.005	0.28 ± 0.011	0.21 ± 0.006	0.19 ± 0.036
C20:5n3	17.69 ± 0.873	16.24 ± 0.003	16.85 ± 0.633	17.90 ± 0.414	17.39 ± 0.073	19.74 ± 0.580	19.38 ± 0.425	16.13 ± 0.831	16.67 ± 0.371	17.90 ± 0.948	19.01 ± 0.228	16.71 ± 1.059
C22:0	0.15 ± 0.024	2.64 ± 2.513	2.69 ± 2.496	0.12 ± 0.002	0.20 ± 0.055	0.31 ± 0.224	0.10 ± 0.001	0.12 ± 0.024	0.63 ± 0.014	0.14 ± 0.035	0.23 ± 0.037	0.08 ± 0.009
C22:1n9	0.19 ± 0.026	0.16 ± 0.010	0.17 ± 0.016	0.18 ± 0.008	0.21 ± 0.016	0.19 ± 0.037	0.15 ± 0.021	0.17 ± 0.023	0.20 ± 0.004	0.18 ± 0.034	0.14 ± 0.020	0.09 ± 0.027
C22:2	0.47 ± 0.014	0.54 ± 0.123	0.55 ± 0.109	0.45 ± 0.011	0.37 ± 0.028	0.39 ± 0.003	0.36 ± 0.015	0.35 ± 0.018	0.40 ± 0.009	0.31 ± 0.014	0.24 ± 0.090	0.31 ± 0.006
C23:0	0.52 ± 0.018	0.48 ± 0.029	0.49 ± 0.015	0.47 ± 0.010	0.45 ± 0.022	0.53 ± 0.034	0.49 ± 0.008	0.53 ± 0.104	0.58 ± 0.013	0.35 ± 0.028	0.37 ± 0.010	0.51 ± 0.100
C22:6n3	7.08 ± 0.665	6.36 ± 0.156	6.58 ± 0.072	6.81 ± 0.169	7.78 ± 0.530	8.60 ± 0.279	8.31 ± 0.198	8.16 ± 1.751	11.33 ± 0.252	7.59 ± 0.300	6.77 ± 0.122	8.58 ± 2.051

**Table 4:** Monthly variation of fatty acid ratios in male individuals of the species *Pontastacus leptodactylus*

	July 2020	August	September	October	November	December	January 2021	February	March	April	May	June
C14:0	0.39 ± 0.001	0.47 ± 0.120	0.31 ± 0.010	0.45 ± 0.023	0.58 ± 0.018	0.52 ± 0.012	0.49 ± 0.014	0.52 ± 0.000	0.43 ± 0.100	0.41 ± 0.107	0.42 ± 0.050	0.40 ± 0.010
C14:1	0.05 ± 0.006	0.08 ± 0.018	0.08 ± 0.012	0.08 ± 0.009	0.08 ± 0.016	0.09 ± 0.017	0.09 ± 0.010	0.10 ± 0.021	0.07 ± 0.011	0.09 ± 0.021	0.09 ± 0.016	0.08 ± 0.007
C15:0	0.51 ± 0.015	0.37 ± 0.180	0.44 ± 0.110	0.38 ± 0.089	0.47 ± 0.018	0.34 ± 0.086	0.36 ± 0.150	0.26 ± 0.091	0.48 ± 0.121	0.57 ± 0.142	0.73 ± 0.026	0.41 ± 0.216
C15:1	0.06 ± 0.016	0.18 ± 0.032	0.12 ± 0.018	0.19 ± 0.012	0.07 ± 0.021	0.07 ± 0.012	0.11 ± 0.000	0.12 ± 0.005	0.08 ± 0.035	0.06 ± 0.005	0.15 ± 0.011	0.15 ± 0.014
C16:0	14.95 ± 0.151	14.40 ± 0.872	15.12 ± 0.451	16.25 ± 0.440	16.83 ± 0.390	17.16 ± 0.727	17.61 ± 0.291	19.37 ± 0.288	19.11 ± 0.386	17.47 ± 1.990	18.16 ± 1.077	16.96 ± 0.442
C16:1	3.33 ± 0.000	3.80 ± 0.116	3.29 ± 0.014	4.86 ± 0.153	5.41 ± 0.101	5.49 ± 0.130	5.20 ± 0.150	4.93 ± 0.065	4.35 ± 0.596	4.48 ± 0.959	2.92 ± 0.366	3.33 ± 0.080
C17:0	0.64 ± 0.004	0.80 ± 0.117	0.72 ± 0.018	0.85 ± 0.030	0.86 ± 0.008	0.78 ± 0.012	0.76 ± 0.002	0.76 ± 0.012	0.77 ± 0.018	0.86 ± 0.004	0.70 ± 0.031	0.45 ± 0.143
C17:1	0.24 ± 0.079	0.25 ± 0.035	0.39 ± 0.030	0.23 ± 0.132	0.27 ± 0.003	0.27 ± 0.010	0.22 ± 0.028	0.35 ± 0.004	0.36 ± 0.003	0.31 ± 0.097	0.21 ± 0.058	0.22 ± 0.041
C18:0	6.05 ± 0.095	4.18 ± 0.621	5.25 ± 0.791	4.42 ± 0.055	4.49 ± 0.219	4.56 ± 0.414	4.67 ± 0.337	6.20 ± 0.265	6.59 ± 0.493	7.14 ± 1.435	7.78 ± 0.026	5.97 ± 0.147
C18:1n9	24.66 ± 0.162	24.76 ± 3.104	25.36 ± 1.950	25.20 ± 1.557	24.65 ± 1.242	23.84 ± 1.900	23.74 ± 1.573	22.43 ± 1.778	23.71 ± 1.617	22.79 ± 1.834	24.46 ± 2.383	24.64 ± 2.088
C18:2n6c	3.58 ± 0.021	4.07 ± 0.180	3.50 ± 0.049	3.85 ± 0.119	3.24 ± 0.075	2.91 ± 0.129	2.87 ± 0.083	2.72 ± 0.046	2.54 ± 0.190	3.78 ± 0.498	3.10 ± 0.153	6.27 ± 0.195
C18:3n6	0.97 ± 0.019	0.99 ± 0.108	0.84 ± 0.028	1.08 ± 0.024	0.97 ± 0.025	1.01 ± 0.049	0.95 ± 0.036	0.98 ± 0.021	0.91 ± 0.064	1.24 ± 0.220	0.91 ± 0.008	0.78 ± 0.044
C18:3n3	0.26 ± 0.001	0.15 ± 0.011	0.13 ± 0.006	0.23 ± 0.002	0.21 ± 0.001	0.17 ± 0.001	0.12 ± 0.016	0.11 ± 0.005	0.09 ± 0.033	0.12 ± 0.045	0.15 ± 0.016	0.04 ± 0.044
C20:0	0.22 ± 0.005	0.13 ± 0.030	0.16 ± 0.039	0.10 ± 0.013	0.09 ± 0.008	0.15 ± 0.009	0.14 ± 0.010	0.11 ± 0.003	0.14 ± 0.024	0.36 ± 0.059	0.30 ± 0.027	0.43 ± 0.015
C20:1n9	1.73 ± 0.014	1.13 ± 0.315	1.68 ± 0.112	1.30 ± 0.190	1.44 ± 0.035	1.35 ± 0.117	1.46 ± 0.012	1.38 ± 0.011	1.44 ± 0.036	1.66 ± 0.512	1.42 ± 0.027	1.47 ± 0.028
C20:2	1.27 ± 0.001	0.37 ± 0.367	0.08 ± 0.010	0.10 ± 0.015	0.11 ± 0.003	0.10 ± 0.006	0.50 ± 0.391	0.76 ± 0.015	0.80 ± 0.039	1.26 ± 0.313	1.07 ± 0.016	1.16 ± 0.046
C20:3n6	0.20 ± 0.008	0.21 ± 0.010	0.22 ± 0.007	0.24 ± 0.013	0.22 ± 0.001	0.21 ± 0.002	0.20 ± 0.006	0.19 ± 0.009	0.18 ± 0.005	0.17 ± 0.022	0.21 ± 0.018	0.21 ± 0.017
C21:0	0.14 ± 0.013	0.09 ± 0.060	0.10 ± 0.021	0.07 ± 0.009	0.11 ± 0.022	0.12 ± 0.036	0.11 ± 0.013	0.07 ± 0.014	0.10 ± 0.027	0.08 ± 0.004	0.11 ± 0.010	0.08 ± 0.006
C20:4n6	12.95 ± 0.084	14.10 ± 0.693	15.00 ± 0.112	12.31 ± 0.155	12.49 ± 0.315	10.44 ± 0.259	10.88 ± 0.360	8.84 ± 0.154	9.15 ± 0.257	10.28 ± 0.941	10.90 ± 0.174	10.39 ± 0.295
C20:3n3	0.19 ± 0.008	0.16 ± 0.010	0.21 ± 0.007	0.19 ± 0.013	0.19 ± 0.000	0.19 ± 0.018	0.22 ± 0.004	0.22 ± 0.005	0.25 ± 0.031	0.27 ± 0.016	0.26 ± 0.011	0.19 ± 0.036
C20:5n3	17.86 ± 0.122	19.33 ± 2.597	17.74 ± 0.029	16.41 ± 0.224	15.68 ± 0.370	17.29 ± 0.263	17.00 ± 0.874	16.51 ± 0.298	16.58 ± 0.004	17.84 ± 0.853	17.37 ± 0.223	17.64 ± 0.447
C22:0	0.16 ± 0.019	0.17 ± 0.028	0.13 ± 0.017	0.18 ± 0.039	0.17 ± 0.022	0.15 ± 0.028	0.15 ± 0.016	0.22 ± 0.013	0.41 ± 0.207	0.20 ± 0.053	0.13 ± 0.025	0.15 ± 0.012
C22:1n9	0.13 ± 0.001	0.37 ± 0.244	0.18 ± 0.022	0.42 ± 0.018	0.32 ± 0.013	0.85 ± 0.509	0.38 ± 0.167	0.51 ± 0.322	0.12 ± 0.079	0.13 ± 0.036	0.22 ± 0.028	0.19 ± 0.021
C22:2	0.50 ± 0.001	0.55 ± 0.016	0.55 ± 0.025	0.58 ± 0.030	0.55 ± 0.055	0.55 ± 0.044	0.46 ± 0.002	0.38 ± 0.003	0.37 ± 0.026	0.40 ± 0.090	0.29 ± 0.027	0.36 ± 0.006
C23:0	0.82 ± 0.006	0.58 ± 0.006	0.59 ± 0.043	0.66 ± 0.022	0.66 ± 0.052	0.72 ± 0.015	0.70 ± 0.037	0.66 ± 0.093	0.56 ± 0.018	0.47 ± 0.038	0.42 ± 0.005	0.42 ± 0.017
C22:6n3	8.15 ± 0.050	8.31 ± 0.379	7.81 ± 0.104	9.36 ± 0.157	9.87 ± 0.250	10.69 ± 0.151	10.59 ± 0.413	11.30 ± 0.272	10.42 ± 0.822	7.56 ± 1.037	7.50 ± 0.151	7.60 ± 0.267

The Monounsaturated Fatty Acid (MUFA) values in female individuals were lowest in March (28.77 ± 1.566%) and highest in August (32.47 ± 2.157%). For male individuals, the lowest MUFA value was recorded in May (29.48 ± 1.956%) and the highest in October (%32.29 ± 1.066). There was no statistically significant difference in MUFA levels between months (p > 0.05). Oleic acid (C18:1n9) and palmitoleic acid (C16:1) were found to be the most abundant MUFAs in both female and male individuals, with average percentages of 23.77 ± 1.179 and 24.19 ± 0.905 for females, and 4.81 ± 0.919 and 4.28 ± 0.916 for males, respectively.

The PUFA (Polyunsaturated Fatty Acid) values of female individuals were found to be the lowest in April (40.99 ± 1.926%) and the highest in October (44.61 ± 1.166%). For male individuals, the lowest PUFA values were recorded in March (41.53 ± 0.787%), while the highest values were observed in August (48.40 ± 4.290%). Among the months, only the difference between October and August was found to be statistically significant (p < 0.05), while the differences between other months were not statistically significant (p > 0.05). The dominant PUFA for both female and male individuals were the ω -3 fatty acids Eicosapentaenoic Acid (EPA C20:5n3) (average %; 17.63 ± 1.213, 17.27 ± 0.937) and Docosahexaenoic Acid (DHA C22:6n3) (average %; 7.83 ± 1.358, 9.10 ± 1.425), along with the omega-6 fatty acid Arachidonic Acid (ARA C20:4n6) (average %; 9.93 ± 1.277, 11.48 ± 1.905).

Lazarević et al. (2022) reported SFA, MUFA, and PUFA values of 27.34%, 26.90%, and 45.76%, respectively, in the Invasive Crayfish species *Faxonius limosus* collected from the Danube River in Serbia, which were similar to the results obtained in this study. Additionally, in the same study, they reported that the dominant PUFA was comprised of ω -3 fatty acids EPA and DHA, along with omega-6 fatty acid ARA, which aligns with the findings of our study.

In the context of crustacean biochemistry, it has been observed that factors such as sex, reproductive period (Buckup et al. 2008), environmental parameters like habitat, feeding activity, and food availability (Buckup et al. 2008), as well as seasonality (Bahadır Koca and Argun Uzunmehmetoğlu 2018), can influence their metabolism and biochemical composition. Lipids, as the main organic reserve in many crustaceans, play a significant role in the metabolic processes of these species. Fatty acids, a component of these lipid reserves utilized as an energy source, are crucial for crustacean species' growth rate, survival rate, reproduction cycle, and molting (Beder et al. 2018; Li et al. 2021). The variations in fatty acids are influenced by factors such as species, gender, environmental parameters (especially temperature), and feeding (Bascur et al. 2017). Crustaceans store lipids and fatty acids in different organs, including the hepatopancreas, viscera, muscle tissue (Guzmán-Rivas et al. 2021), and gills (Wang et al. 2007). The hepatopancreas is considered the primary organ for lipid storage and digestion in crustaceans.

According to McLay and van den Brink (2016), growth and reproductive activities of crayfish vary seasonally. For *P. leptodactylus* species, reproductive activity usually begins in autumn when the water temperature starts to decrease, with mating occurring in October–November when the water temperature ranges between 7–12 °C. Egg-laying takes place 4 to 6 weeks later at water temperatures of 6–11 °C. *P. leptodactylus* reproduces only once a year, has low fertility, and undergoes a long embryonic development period (6–9 months) under natural conditions (Reynolds et al. 1992). During reproductive activity and molting, it is expected that energy reserves are utilized more efficiently (Nguyen et al. 2022). Oliveira et al. (2007) reported increased energy costs for gamete production in the summer, incubation and egg-laying in autumn and winter, and parental care in spring and summer for the *Aegla platensis* species. In this study, we observed that both female and male individuals exhibited variability in abdominal lipid and fatty acid content over the months. We found that eggs were present internally between August and December, and externally between January and April. Wu and Wang (2017) reported that after female and male crabs (*Eriocheir sinensis*) completed ovarian maturation, their lipid levels remained stable. However, a clear relationship between reproductive activity and changes in abdominal fat and fatty acid storage could not be established, and no consistent pattern was identified. Therefore, this variability can be attributed to 1) reproductive activities, growth, and molting processes, 2) the prioritization of energy storage in the hepatopancreas, with the abdomen being used as needed, and 3) direct acquisition of energy from food.

Gender in crustaceans can also contribute to variations in biochemical composition and fatty acid composition, as females require more energy for the development of oocytes, incubation, egg-laying, and parental care (Rosa and Nunes 2003; Wu et al. 2010; Tufan 2022). Sun et al. (2023)found that males had significantly higher meat yield and lower crude fat content in the abdomen compared to females. Females in crustaceans generally need to utilize more energy than males for these reproductive processes. This energy can be derived from food intake, different tissue reserves, or a combination of both (Buckup et al. 2008). In our study, we observed that lipid content changed with months, with the lowest levels found in October for females and November for males. Overall, males had higher lipid content than females, except in September. Some studies have reported the opposite for crayfish, where females were found to have higher total lipid content (Bahadır Koca and Argun Uzunmehmetoğlu 2018). However, Mona et al. (2000) found higher fat levels in male individuals and attributed this to the demand for these materials during the active metabolic processes required for the rapid completion of their life cycle. Similarly, in this study, the lower lipid content in female individuals might be due to their prolonged gonad maturation, egg-laying, and care phases, leading to a higher energy demand.

The distinctive feature of the nutritional value of seafood lies in the dominance of essential long-chain polyunsaturated fatty acids (PUFAs). The health benefits of *P. leptodactylus* can be assessed using lipid quality indices such as PUFA/SFA ratio, ω -6/ ω -3 ratio, atherogenic index (AI), thrombogenic index (TI), and absolute EPA + DHA amounts (mg/100 g edible portion). The lipid profiles of *P. leptodactylus* are presented in Table 5.

**Table 5:** Monthly assessment of fatty acid quality ratios in both female and male individuals of the species *Pontastacus leptodactylus*

		July 2020	August	September	October	November	December	January 2021	February	March	April	May	June
Sfa	♂	23.87 ± 0.082^abc^	21.18 ± 1.324^a^	22.82 ± 1.500^ab^	23.37 ± 0.375^ab^	24.26 ± 0.133^abc^	24.49 ± 1.297^abcd^	25.00 ± 0.129^abcde^	28.17 ± 0.540^de^	28.60 ± 0.118^e^	27.55 ± 3.422^bcde^	28.75 ± 1.216^e^	25.28 ± 0.713^bcde^
	♀	23.67 ± 0.040^ab^	25.14 ± 2.112^abc^	24.73 ± 2.585^abc^	23.25 ± 1.230^a^	27.44 ± 4.284^abc^	25.81 ± 0.662^abc^	26.27 ± 0.639^abc^	27.46 ± 1.396^abc^	28.88 ± 0.624^bc^	29.09 ± 1.214^c^	27.88 ± 1.144^abc^	27.81 ± 1.368^abc^
mufa	♂	30.20 ± 0.218^a^	30.58 ± 2.975^a^	31.10 ± 1.785^a^	32.29 ± 1.066^a^	32.22 ± 1.117^a^	31.96 ± 2.124^a^	31.21 ± 1.262^a^	29.83 ± 1.350^a^	30.13 ± 0.936^a^	29.53 ± 3.199^a^	29.48 ± 1.956^a^	30.08 ± 2.021^a^
	♀	32.05 ± 1.337^a^	32.47 ± 2.157^a^	32.45 ± 2.177^a^	32.39 ± 2.397^a^	30.91 ± 3.260^a^	30.23 ± 1.854^a^	30.42 ± 1.550^a^	31.47 ± 2.746^a^	28.77 ± 1.566^a^	30.20 ± 3.129^a^	28.59 ± 2.032^a^	29.51 ± 3.331^a^
pufa	♂	46.12 ± 0.292^bc^	48.40 ± 4.290^c^	46.30 ± 0.292^bc^	44.53 ± 0.703^abc^	43.70 ± 0.984^ab^	43.75 ± 0.844^ab^	44.02 ± 1.387^ab^	42.22 ± 0.816^ab^	41.53 ± 0.787^a^	43.19 ± 0.207^ab^	42.03 ± 0.729^ab^	44.84 ± 1.344^abc^
	♀	44.53 ± 1.365^ab^	42.67 ± 0.089^ab^	43.11 ± 0.369^ab^	44.61 ± 1.166^b^	41.89 ± 0.977^ab^	44.24 ± 1.196^ab^	43.58 ± 0.918^ab^	41.25 ± 1.384^ab^	42.57 ± 0.947^ab^	40.99 ± 1.926^a^	43.74 ± 0.894^ab^	42.88 ± 2.000^ab^
omega 3	♂	26.45 ± 0.166^a^	27.94 ± 2.978^a^	25.90 ± 0.076^a^	26.19 ± 0.396^a^	25.94 ± 0.620^a^	28.34 ± 0.430^a^	27.93 ± 1.299^a^	28.14 ± 0.570^a^	27.33 ± 0.827^a^	25.79 ± 1.862^a^	25.28 ± 0.380^a^	25.48 ± 0.706^a^
	♀	25.17 ± 1.503^abc^	22.98 ± 0.195^a^	23.81 ± 0.674^a^	25.06 ± 0.577^abc^	25.51 ± 0.623^abc^	28.73 ± 0.871^c^	27.98 ± 0.600^bc^	24.63 ± 2.594^ab^	28.34 ± 0.630^bc^	25.86 ± 1.266^abc^	26.14 ± 0.353^abc^	25.62 ± 3.109^abc^
omega 6	♂	17.70 ± 0.132^def^	19.37 ± 0.971^ef^	19.56 ± 0.195^f^	17.47 ± 0.310^d^	16.92 ± 0.416^ cd^	14.56 ± 0.434^ab^	14.91 ± 0.485^b^	12.73 ± 0.229^a^	12.78 ± 0.003^a^	15.47 ± 1.681^bc^	15.13 ± 0.317^bc^	17.65 ± 0.551^de^
	♀	17.18 ± 0.011^e^	17.62 ± 0.224^e^	17.22 ± 0.186^e^	17.54 ± 0.532^e^	14.69 ± 1.449^abcd^	13.79 ± 0.318^abc^	13.90 ± 0.269^abc^	14.99 ± 1.162^bcd^	12.85 ± 0.286^a^	13.46 ± 0.592^ab^	16.09 ± 0.402^de^	15.62 ± 1.065^cde^
omega 9	♂	24.79 ± 0.163^a^	25.13 ± 2.861^a^	25.54 ± 1.972^a^	25.62 ± 1.538^a^	24.97 ± 1.255^a^	24.69 ± 2.409^a^	24.13 ± 1.406^a^	22.94 ± 1.456^a^	23.83 ± 1.538^a^	22.92 ± 1.799^a^	24.68 ± 2.411^a^	24.83 ± 2.067^a^
	♀	23.94 ± 1.259^a^	24.71 ± 2.240^a^	25.04 ± 2.616^a^	25.07 ± 2.773^a^	24.45 ± 2.123^a^	22.21 ± 2.017^a^	23.49 ± 1.662^a^	24.87 ± 2.723^a^	21.75 ± 1.702^a^	25.18 ± 3.550^a^	23.96 ± 2.308^a^	22.65 ± 3.282^a^
omega 6/3	♂	0.67 ± 0.001	0.70 ± 0.038	0.76 ± 0.005	0.67 ± 0.002	0.65 ± 0.000	0.51 ± 0.008	0.53 ± 0.007	0.45 ± 0.001	0.47 ± 0.014	0.61 ± 0.110	0.60 ± 0.004	0.69 ± 0.002
	♀	0.68 ± 0.041	0.77 ± 0.016	0.72 ± 0.028	0.70 ± 0.005	0.58 ± 0.071	0.48 ± 0.003	0.50 ± 0.001	0.62 ± 0.113	0.45 ± 0.000	0.52 ± 0.003	0.62 ± 0.007	0.62 ± 0.116
omega3/6	♂	1.49 ± 0.002	1.44 ± 0.080	1.32 ± 0.009	1.50 ± 0.004	1.53 ± 0.001	1.95 ± 0.028	1.87 ± 0.026	2.21 ± 0.005	2.14 ± 0.064	1.69 ± 0.303	1.67 ± 0.010	1.44 ± 0.005
	♀	1.47 ± 0.088	1.30 ± 0.028	1.38 ± 0.054	1.43 ± 0.010	1.75 ± 0.212	2.08 ± 0.015	2.01 ± 0.004	1.66 ± 0.302	2.21 ± 0.000	1.92 ± 0.010	1.62 ± 0.019	1.65 ± 0.315
DHA/EPA	♂	0.46 ± 0.000	0.43 ± 0.037	0.44 ± 0.007	0.57 ± 0.002	0.63 ± 0.001	0.62 ± 0.001	0.62 ± 0.008	0.68 ± 0.004	0.63 ± 0.049	0.42 ± 0.038	0.43 ± 0.003	0.43 ± 0.004
	♀	0.40 ± 0.018	0.39 ± 0.010	0.39 ± 0.010	0.38 ± 0.001	0.45 ± 0.029	0.44 ± 0.001	0.43 ± 0.001	0.50 ± 0.083	0.68 ± 0.000	0.42 ± 0.006	0.36 ± 0.002	0.51 ± 0.090
UNSFA/SFA	♂	3.20 ± 0.014	3.74 ± 0.304	3.40 ± 0.287	3.29 ± 0.069	3.13 ± 0.023	3.10 ± 0.215	3.01 ± 0.021	2.56 ± 0.068	2.51 ± 0.016	2.68 ± 0.465	2.49 ± 0.149	2.97 ± 0.111
	♀	3.24 ± 0.007	3.01 ± 0.338	3.09 ± 0.432	3.32 ± 0.228	2.72 ± 0.604	2.89 ± 0.099	2.82 ± 0.092	2.65 ± 0.185	2.47 ± 0.075	2.45 ± 0.145	2.60 ± 0.148	2.61 ± 0.175
AI	♂	0.22 ± 0.002	0.21 ± 0.008	0.21 ± 0.010	0.24 ± 0.008	0.25 ± 0.007	0.25 ± 0.013	0.26 ± 0.004	0.30 ± 0.006	0.29 ± 0.012	0.26 ± 0.034	0.28 ± 0.023	0.25 ± 0.009
	♀	0.24 ± 0.002	0.23 ± 0.003	0.22 ± 0.009	0.23 ± 0.015	0.27 ± 0.043	0.26 ± 0.008	0.26 ± 0.008	0.28 ± 0.012	0.30 ± 0.009	0.28 ± 0.019	0.26 ± 0.017	0.30 ± 0.015
h/H	♂	4.49 ± 0.053	4.86 ± 0.310	4.60 ± 0.245	4.12 ± 0.166	3.88 ± 0.103	3.78 ± 0.210	3.68 ± 0.050	3.18 ± 0.095	3.27 ± 0.121	3.62 ± 0.481	3.50 ± 0.305	3.91 ± 0.150
	♀	4.02 ± 0.058	4.16 ± 0.095	4.27 ± 0.207	4.18 ± 0.305	3.59 ± 0.588	3.59 ± 0.121	3.62 ± 0.122	3.40 ± 0.224	3.12 ± 0.109	3.42 ± 0.258	3.74 ± 0.285	3.26 ± 0.221
TI	♂	0.15 ± 0.001	0.13 ± 0.017	0.14 ± 0.009	0.15 ± 0.003	0.15 ± 0.001	0.16 ± 0.007	0.16 ± 0.005	0.19 ± 0.002	0.19 ± 0.002	0.19 ± 0.023	0.20 ± 0.008	0.18 ± 0.000
	♀	0.16 ± 0.005	0.16 ± 0.002	0.16 ± 0.003	0.16 ± 0.006	0.19 ± 0.035	0.17 ± 0.000	0.18 ± 0.002	0.20 ± 0.007	0.19 ± 0.002	0.21 ± 0.003	0.19 ± 0.008	0.20 ± 0.009
HPI	♂	1.45 ± 0.009	1.30 ± 0.050	1.39 ± 0.050	1.29 ± 0.017	1.27 ± 0.024	1.27 ± 0.022	1.28 ± 0.029	1.31 ± 0.008	1.37 ± 0.046	1.44 ± 0.062	1.45 ± 0.032	1.36 ± 0.003
	♀	1.31 ± 0.016	1.47 ± 0.146	1.47 ± 0.146	1.33 ± 0.003	1.41 ± 0.077	1.31 ± 0.007	1.36 ± 0.003	1.33 ± 0.036	1.33 ± 0.001	1.46 ± 0.014	1.50 ± 0.015	1.30 ± 0.020
FLQ	♂	10.93 ± 0.072	6.47 ± 0.697	8.72 ± 0.026	12.57 ± 0.186	22.61 ± 0.549	10.52 ± 0.156	9.96 ± 0.465	7.47 ± 0.153	5.12 ± 0.157	7.08 ± 0.527	5.84 ± 0.088	6.84 ± 0.194
	♀	11.31 ± 0.702	5.95 ± 0.042	7.14 ± 0.215	32.52 ± 0.767	15.63 ± 0.374	14.31 ± 0.434	5.74 ± 0.129	5.19 ± 0.552	5.96 ± 0.133	5.83 ± 0.286	5.37 ± 0.073	10.12 ± 1.244
PI	♂	1.74 ± 0.029	1.93 ± 0.333	1.69 ± 0.045	1.59 ± 0.020	1.52 ± 0.002	1.63 ± 0.045	1.57 ± 0.047	1.44 ± 0.008	1.41 ± 0.015	1.46 ± 0.059	1.37 ± 0.061	1.49 ± 0.003
	♀	1.56 ± 0.064	1.49 ± 0.026	1.56 ± 0.047	1.57 ± 0.038	1.43 ± 0.139	1.62 ± 0.016	1.57 ± 0.001	1.32 ± 0.070	1.43 ± 0.000	1.38 ± 0.001	1.48 ± 0.058	1.33 ± 0.092
pufa/safa	♂	1.93 ± 0.019	2.30 ± 0.356	2.03 ± 0.120	1.91 ± 0.001	1.80 ± 0.031	1.79 ± 0.060	1.76 ± 0.065	1.50 ± 0.000	1.45 ± 0.022	1.58 ± 0.208	1.46 ± 0.037	1.77 ± 0.003
	♀	1.88 ± 0.061	1.71 ± 0.141	1.76 ± 0.202	1.92 ± 0.051	1.56 ± 0.291	1.71 ± 0.002	1.66 ± 0.005	1.50 ± 0.026	1.47 ± 0.001	1.41 ± 0.007	1.57 ± 0.033	1.54 ± 0.004
EFA	♂	618.94	1180.12	748.70	528.23	288.70	744.31	764.20	1034.40	1422.58	911.83	1059.49	931.67
	♀	542.51	858.50	768.36	187.81	405.26	561.08	1334.48	1136.68	1316.07	1114.07	1237.28	632.44

^ab^ ( →): The difference between the mean values shown in different letters at each month level is statistically significant (*P* < 0.05)

A crucial indicator of lipid quality in seafood is the PUFA/SFA ratio, which reflects the effects of both PUFAs and SFAs, thus representing a balanced fatty acid composition. High SFA intake (and consequently a lower PUFA/SFA ratio) has been shown to increase the risk of cardiovascular health issues (Vissers et al. 2018; Markey et al. 2014). In our study, PUFA/SFA ratios ranged from 1.41 to 1.92 in female individuals and from 1.45 to 2.30 in male individuals. As the recommended PUFA/SFA ratio falls within the range of 0.45–4.00, both female and male *P. leptodactylus* can be considered a healthy food choice. The UNSAFA/SAFA ratio for female individuals was found to be between 2.45 and 3.32, while for male individuals, it ranged from 2.49 to 3.74.

Maintaining a balance between ω -3 and ω -6 fatty acid intake is recommended for preserving health. Additionally, it is advised to avoid excessive omega-6 intake while increasing ω -3 consumption to reduce the risk of obesity and cardiovascular diseases (Simopoulos 2002). In our study, the lowest levels of ω -3 fatty acids were observed in August (22.98 ± 0.195%) for female individuals and in May (25.28 ± 0.380%) for male individuals. The highest levels of ω -3 were recorded in December (28.73 ± 0.871%) for female individuals and in December (28.34 ± 0.430%), similar to female individuals, for male individuals. No statistically significant difference was found among the months (p > 0.05). Moreover, the lowest omega-6 value for female individuals was noted in March (12.85 ± 0.286%), and the highest was in August (17.62 ± 0.22%). For male individuals, the lowest ω -6 value was observed in February (12.78 ± 0.003%), while the highest was in September (19.56 ± 0.195%), with a statistically significant difference between months (p < 0.05).

Historically, the ω-6:ω-3 ratio was 1:1 in ancient human diets. With industrialization and changes in dietary patterns, this ratio has increased to 30:1 to 50:1. The World Health Organization recommends an ω-6:ω-3 ratio between 5:1 and 10:1 (FAO/WHO 1994). However, for a healthy diet, this ratio should be between 1:1 and 1:4 (Simopoulos et al. 2000). In our study, the ω-6:ω-3 ratio for female individuals was found to be the lowest in August (1:1.30) and the highest in March (1:2.21). For male individuals, the lowest ratio was observed in September (1:1.32), while the highest was in February (1:2.21). It is evident that *P. leptodactylus* is an extremely healthy food choice and can be included in a balanced diet. Lazarević et al. (2022) reported a similar ω-6:ω-3 ratio of 1:1.79, while Śmietana et al. (2020) reported a ratio of 1:1.08 for spiny-cheek crayfish collected from Lake Sominko in Poland.

According to dietary guidelines, a dosage of 250–500 mg of long-chain omega-3 fatty acids EPA + DHA per week (equivalent to one to two servings) is recommended for human health. In our study, both female and male *P. leptodactylus* showed remarkably high average values for EPA + DHA (841.21 mg/100 g EP for females and 852.76 mg/100 g EP for males).

We examined the fatty acid values of *A. leptodactylus* on a monthly basis. In this study, the Fatty Acid Quality (FLQ) index, which indicates the increase in dietary quality as the FLQ index increases, showed similar results to the study conducted by Abrami et al. (1992). The lowest FLQ value for female individuals was recorded in October (5.19), and the highest FLQ value was observed in February (32.52). For male individuals, the lowest FLQ value was found in March (5.12), and the highest value was in November (22.61). According to Bentes et al. (2009), the h/H ratio of fatty acids indicates whether the fat in the diet is sufficient or not. In this study, the lowest h/H ratio for female individuals was observed in March (3.12), and the highest h/H ratio was in September (4.27). For male individuals, the lowest h/H ratio was recorded in February (3.18), and the highest was in August (4.86). Śmietana et al. (2020) reported a similar h/H ratio of 3.30 in their study. In Bayrakli’s (2021a) crab study, the h/H values ranged from 2.45 to 4.20. Based on these findings, it can be concluded that the h/H value of freshwater crayfish meat is comparable to crab meat values.

A decrease in the PI (Proportionality Index) indicates a deterioration in PUFA quality (Šimat et al. 2015). In this study, the lowest PI value in female individuals was in February (1.49), and the highest was in December (1.62). In male individuals, the lowest PI value was in May (1.37), and the highest was in August (1.93). The PI values in this study are similar to those reported by Bayrakli (2021a) for crab meat (ranging from 1.18 to 2.53).

The potential health benefits of freshwater crayfish were evaluated based on two additional indices: the Atherogenic Index (AI) and the Thrombogenic Index (TI) which are reported to be harmful to humans if they exceed 1.0 (Ouraji et al. 2009). Higher AI and TI values can stimulate platelet aggregation and thrombus formation, thus lower values are considered beneficial for human health. In this study, the lowest AI value for female individuals was observed in September (0.22), while the highest was in March (0.30). For male individuals, the highest AI value was recorded in February (0.30), and the lowest in August (0.21). Furthermore, the highest TI value for female individuals was observed in September (0.21), while the lowest was in April (0.16). In male individuals, the lowest TI value was found in August (0.13), and the highest in May (0.20). Lazarević et al. (2022) reported similar AI and TI values of 0.40 and 0.21, respectively, in their study. Śmietana et al. (2020) also reported an AI and TI value of 0.29. Bayraklı (2021a) reported AI values ranging from 0.25 to 0.43, and TI values ranging from 0.16 to 0.30 in their crab study.

Based on these results, it can be concluded that the meat of both female and male freshwater crayfish is rich in healthy and high-quality fatty acids, similar to other seafood. Therefore, consuming freshwater crayfish may be highly beneficial for reducing the risk of cardiovascular diseases.

### Evaluation of benefit-risk ratio associated with *P. leptodactylus* and its potential impact on human health

The values of benefit-risk hazard quotients (HQEFA) are presented in Table 6. Upon examination, female *P. leptodactylus* specimens showed high HQEFA values (> 1) for the Cu element in October, while both male and female specimens exhibited elevated HQEFA values for Hg elements, indicating potential risks for consumers. However, it should be noted that the recommended intake of EPA + DHA (500 mg per day) is met through the consumption of *P. leptodactylus*. Consequently, the benefits of EPA + DHA obtained from consuming *P. leptodactylus* outweigh the associated risks.

**Table 6:** Monthly metal and fatty acid beneficial utilization table for both female and male individuals of the species *Pontastacus leptodactylus*

		fe	Cu	Mn	Zn	Pb	Cd	Se	As	Hg	Al
July-2020	♀	0.04	0.48	0.02120	0.08	0.00	0.04	0.09	0.03	0.78	0.02
	♂	0.02	0.36	0.02292	0.07	0.00	0.02	0.08	0.03	0.37	0.02
August	♀	0.01	0.28	0.00697	0.05	0.01	0.01	0.05	0.02	0.48	0.01
	♂	0.03	0.15	0.00377	0.04	0.01	0.01	0.04	0.01	0.31	0.00
September	♀	0.03	0.37	0.02599	0.06	0.01	0.02	0.06	0.02	0.79	0.05
	♂	0.17	0.38	0.03384	0.06	0.04	0.04	0.07	0.03	0.92	0.10
October	♀	0.15	**1.49**	0.07048	0.24	0.02	0.10	0.32	0.09	**3.13**	0.26
	♂	0.07	0.40	0.02035	0.10	0.02	0.03	0.09	0.03	**1.41**	0.14
November	♀	0.05	0.65	0.02161	0.10	0.00	0.04	0.14	0.03	**1.09**	0.08
	♂	0.23	0.79	0.05669	0.17	0.04	0.06	0.18	0.07	**2.43**	0.23
December	♀	0.13	0.55	0.06920	0.10	0.01	0.03	0.10	0.02	0.66	0.08
	♂	0.06	0.35	0.04306	0.08	0.02	0.02	0.08	0.03	0.62	0.08
January-21	♀	0.03	0.17	0.01267	0.04	0.00	0.01	0.04	0.01	0.28	0.02
	♂	0.04	0.30	0.02394	0.08	0.00	0.01	0.08	0.03	0.49	0.02
February	♀	0.01	0.30	0.02264	0.06	0.02	0.02	0.06	0.02	0.39	0.02
	♂	0.01	0.17	0.01087	0.05	0.02	0.01	0.05	0.01	0.34	0.01
March	♀	0.01	0.16	0.01379	0.03	0.00	0.01	0.04	0.01	0.23	0.02
	♂	0.03	0.13	0.01334	0.03	0.00	0.01	0.04	0.01	0.21	0.02
April	♀	0.02	0.23	0.01878	0.04	0.01	0.01	0.04	0.01	0.44	0.02
	♂	0.01	0.27	0.01624	0.06	0.00	0.01	0.07	0.02	0.37	0.01
May	♀	0.01	0.25	0.01277	0.04	0.00	0.02	0.04	0.01	0.60	0.01
	♂	0.01	0.22	0.01186	0.05	0.00	0.01	0.05	0.01	0.32	0.02
June	♀	0.04	0.35	0.01388	0.08	0.02	0.02	0.07	0.03	0.85	0.02
	♂	0.01	0.20	0.00483	0.05	0.00	0.01	0.05	0.02	0.42	0.00

Upon examination of EDI values, it was found that all elements’ values were below the RfD level for both adult and child groups (Table 7). THQ is considered a reasonable parameter for the risk assessment of seafood consumption with metal contamination (Bogdanović et al. 2014). If the THQ value is below 1, the exposed population is not expected to experience adverse effects, while a THQ value above 1 indicates a potential risk for non-carcinogenic effects, as previously reported by Bayrakli (2021b). In this study, THQ values for all metals were found to be well below 1 in both groups. The TTHQ, representing the cumulative effect of multiple metals in crayfish meat, was found to be less than 1. In this study, CRR cancer risk values for As and Pb in both adult and child groups were found to be negligible (< 10^–7^).

**Table 7:** Health risk analysis for consumption of male and female *Pontastacus leptodactylus* individuals based on metal results, considering adult and child human populations"

Metals		RfD mg kg^−1^ ww	EDI (mg kg^−1^ww)		CR_lim_ (kg)		Meal/Day		THQ		CR		EAR (mg/day, person ww)	CDRmin	
			Adult	Child	Adult	Child	Adult	Child	Adult	Child	Adult	Child			
Al	♀	1	9 × 10^–4^	**2 × 10**^**–3**^	2.077	1.068	> 5	> 5	9 × 10^–4^	2 × 10^–3^					
	♂		1 × 10^–3^	3 × 10^–3^	1.273	0.6555	> 5	> 5	1 × 10^–3^	3 × 10^–3^					
As	♀	3 × 10^–4^	2 × 10^–7^	4 × 10^–7^	2.692	1.385	> 5	> 5	7 × 10^–4^	1 × 10^–3^	3 × 10^–7^	6 × 10^–7^			
	♂		3 × 10^–7^	6 × 10^–7^	2.000	1.029	> 5	> 5	1 × 10^–3^	2 × 10^–3^	4 × 10^–7^	8 × 10^–7^			
Cd	♀	1 × 10^–4^	1 × 10^–6^	2 × 10^–6^	1.750	0.900	> 5	> 5	2.6 × 10^–2^	5.2 × 10^–2^					
	♂		1 × 10^–6^	2 × 10^–6^	1.750	0.900	> 5	> 5	1 × 10^–2^	2 × 10^–2^					
Cu	♀	0,04	5 × 10^–4^	1 × 10^–3^	**0.145**	**0.075**	**0.64**	**0.65**	0.013	0.025			0.700	16.99	21.50
	♂		4 × 10^–4^	8 × 10^–4^	**0.176**	**0.091**	**0.78**	**0.79**	0.011	0.021				13.98	17.70
Fe	♀	0,7	2 × 10^–3^	4 × 10^–3^	0.687	0.354	> 3	> 3	3 × 10^–3^	5 × 10^–3^			6,00	7.32	7.39
	♂		3 × 10^–3^	6 × 10^–3^	0.398	0.205	> 1	> 1	5 × 10^–3^	9 × 10^–3^				12.63	12.80
Hg	♀	1 × 10^–4^	3 × 10^–6^	5 × 10^–6^	**0.070**	**0.036**	**0.31**	**0.32**	0.027	0.053					
	♂		3 × 10^–6^	5 × 10^–6^	**0.070**	**0.036**	**0.31**	**0.32**	0.027	0.053					
Mn	♀	0,14	2 × 10^–4^	4 × 10^–4^	1.288	0.662	> 5	> 5	1 × 10^–3^	3 × 10^–3^			2.30	2.04	2.62
	♂		2 × 10^–4^	3 × 10^–4^	1.560	0.803	> 5	> 5	1 × 10^–3^	2 × 10^–3^				1.68	2.16
Pb	♀	4 × 10^–3^	1 × 10^–6^	3 × 10^–6^	2.857	1.469	> 5	> 5	7 × 10^–4^	1 × 10^–3^	1 × 10^–8^	2 × 10^–8^			
	♂		2 × 10^–6^	4 × 10^–6^	1.944	1.000	> 5	> 5	1 × 10^–3^	2 × 10^–3^	2 × 10^–8^	3 × 10^–8^			
Se	♀	0,005	1 × 10^–5^	3 × 10^–5^	0.729	0.375	> 3	> 3	3 × 10^–3^	5 × 10^–3^			0.045	6.57	8.25
	♂		1 × 10^–5^	2 × 10^–5^	0.814	0.419	> 3	> 3	2 × 10^–3^	5 × 10^–3^				5.89	7.39
Zn	♀	0,3	7 × 10^–4^	1 × 10^–3^	0.798	0.410	> 3	> 3	2 × 10^–3^	5 × 10^–3^			9.40	1.73	2.26
	♂		7 × 10^–4^	1 × 10^–3^	0.852	0.438	> 3	> 3	2 × 10^–3^	4 × 10^–3^				1.62	2.12
TTHQ									0.054	0.104					
Monthly Cu	♀				4.352	2.238	19.17	19.63							
	♂				5.289	2.720	23.30	23.86							
Monthly Hg	♀				2.100	1.08	9.25	9.47							
	♂				2.100	1.08	9.25	9.47							

To determine the meal quantities that can be consumed without cancer risk through food intake, cancer risk levels (CR_lim_) were calculated (US-EPA 2023a). In the study, CR_lim_ values for metals other than Hg in adults and children did not indicate any adverse conditions that would affect the daily consumption limits of crayfish meat. For Hg, no issues were detected in terms of Edi, THQ, and CRR, and the Meal/Day values remained below one portion, with CR_lim_ being determined as 0.070 kg for adults and 0.036 kg for children, respectively. While Se is believed to mitigate Hg toxicity, it cannot entirely prevent it. It is important to note that avoiding environmental Hg pollution, tracing the sources of consumed fish and seafood, and maintaining a healthy diet are equally crucial in minimizing health risks. Additionally, excessive Se intake can also pose potential risks, and high Se levels can enhance the bioavailability of Hg compounds by reacting with Hg. However, Hg, being a toxic metal, can pose serious health risks to humans, and high levels of Hg in crayfish meat can lead to toxicity. Specifically, daily consumption of crayfish meat may create a cancer risk for human health due to elevated Hg levels. Nevertheless, when considering meal/month calculations involving Hg, it was found that the consumption of 4.35 portions by adults and 2.24 portions by children of female crayfish, and 5.29 portions by adults and 2.72 portions by children of male crayfish meat.

Currently, the use of Se–Hg ratios in risk assessment, risk management, or risk communication may still be in its early stages. However, there is a consensus that individuals who consume large amounts of fish with Hg/Se ratios below 1 may face a higher risk of Hg toxicity (Burger and Gochfeld 2011). As the relative amount of Se increases compared to Hg, the risk can be reduced, but the optimal Se level has not yet been determined. This study reveals that the Hg/Se ratios in crayfish samples collected throughout the year ranged from 0.04 to 0.13, well below 1.

Additionally, the Se-HBV is a quality index that facilitates the interpretation of risk and benefit assessments based on Se and Hg levels. A positive Se-HBV indicates health benefits, whereas negative values indicate potential risks. The Se-HBV values also remained positive throughout the year, ranging from 3.96 to 6.01 (Table 8).

**Table 8:** Assessment of health risk for consumption of monthly female and male *Pontastacus leptodactylus* individuals, utilizing the selenium health benefit value (Se-HBV) incorporating Se and Hg levels

		July (2020)	August	September	October	November	December	January (2021)	February	March	April	May	June
Hg/Se	♀	0.07	0.07	0.10	0.08	0.06	0.05	0.05	0.05	0.04	0.08	0.13	0.09
	♂	0.04	0.06	0.10	0.12	0.11	0.06	0.05	0.05	0.04	0.04	0.05	0.06
HBVSe	♀	4.39	4.06	4.25	5.25	4.97	5.08	5.24	6.01	4.82	4.41	3.93	3.96
	♂	4.30	4.57	4.72	4.23	4.51	5.27	5.43	4.56	4.75	5.28	5.00	4.25

According to the information provided in Table 6, the consumption of crayfish by adult female individuals meets approximately 16.99% of their daily basic element needs for copper, 7.32% for iron, 2.04% for manganese, 6.57% for Se, and 1.73% for Zn. For the child group, the corresponding percentages are 21.50%, 7.39%, 2.62%, 8.25%, and 2.26%, respectively. In the case of adult male individuals, the consumption of crayfish meets approximately 13.98% of their copper needs, 12.63% of iron needs, 1.68% of manganese needs, 5.89% of Se needs, and 1.62% of zinc needs. Except for iron, it can be stated that the consumption of crayfish by male individuals helps meet higher amounts of element needs in comparison to the child group.

## Conclusion

Based on the findings, crayfish from both female and male individuals are a safe food source with a balanced fatty acid composition, making them a safety dietary option. The PUFA/SFA ratio falls within recommended ranges, indicating favorable lipid quality. Additionally, the importance of ω-3 and ω-6 fatty acids in reducing the risk of obesity and cardiovascular disease is emphasized. While ω-3 fatty acid content remains consistent across months, ω-6 fatty acid content varies. Overall, crayfish meat contains a balanced ratio of ω-3 and ω-6 fatty acids.

Analyses including the FLQ, h/H ratio of fatty acids, PI, AI, and TI confirm that crayfish meat comprises healthy fatty acids, potentially reducing cardiovascular disease risk.

Trace element analyses revealed high metal content, suggesting a potential risk. However, considering EPA + DHA intake, crayfish consumption is deemed beneficial.

Meal/month calculations show safe consumption levels for adults (4.35 servings/month) and children (2.24 servings/month) of female crayfish, and for males (5.29 servings/month for adults, 2.72 servings/month for children).

These results suggest that crayfish meat, with its moderate Hg levels, can be consumed safely in moderation. However, caution is advised regarding fish and seafood with high Hg content.

In summary, freshwater crayfish can be a valuable nutritional source, but mindful consumption and awareness of trace metal analysis and risk assessment are crucial.

## Data Availability

The datasets used and/or analyzed during the current study are available from the corresponding author on reasonable request.
